# *Streptococcus mutans* Lacking *sufCDSUB* Is Viable, but Displays Major Defects in Growth, Stress Tolerance Responses and Biofilm Formation

**DOI:** 10.3389/fmicb.2021.671533

**Published:** 2021-06-24

**Authors:** Kassapa Ellepola, Xiaochang Huang, Ryan P. Riley, Jacob P. Bitoun, Zezhang Tom Wen

**Affiliations:** ^1^Department of Oral and Craniofacial Biology, School of Dentistry, Louisiana State University Health Sciences Center, New Orleans, LA, United States; ^2^Department of Microbiology, Tulane University, New Orleans, LA, United States; ^3^Department of Microbiology, Immunology and Parasitology, Louisiana State University Health Sciences Center, New Orleans, LA, United States

**Keywords:** *Streptococcus mutans*, iron-sulfur clusters, stress tolerance response, biofilm, oxidative stress

## Abstract

*Streptococcus mutans* appears to possess a sole iron-sulfur (Fe-S) cluster biosynthesis system encoded by the *sufCDSUB* cluster. This study was designed to examine the role of *sufCDSUB* in *S. mutans* physiology. Allelic exchange mutants deficient of the whole *sufCDSUB* cluster and in individual genes were constructed. Compared to the wild-type, UA159, the *sufCDSUB*-deficient mutant, Δ*suf::kan^*r*^*, had a significantly reduced growth rate, especially in medium with the absence of isoleucine, leucine or glutamate/glutamine, amino acids that require Fe-S clusters for biosynthesis and when grown with medium adjusted to pH 6.0 and under oxidative and nitrosative stress conditions. Relative to UA159, Δ*suf::kan^*r*^* had major defects in stress tolerance responses with reduced survival rate of > 2-logs following incubation at low pH environment or after hydrogen peroxide challenge. When compared to UA159, Δ*suf::kan^*r*^* tended to form aggregates in broth medium and accumulated significantly less biofilm. As shown by luciferase reporter fusion assays, the expression of *sufCDSUB* was elevated by > 5.4-fold when the reporter strain was transferred from iron sufficient medium to iron-limiting medium. Oxidative stress induced by methyl viologen increased *sufCDSUB* expression by > 2-fold, and incubation in a low pH environment led to reduction of *sufCDSUB* expression by > 7-fold. These results suggest that lacking of SufCDSUB in *S. mutans* causes major defects in various cellular processes of the deficient mutant, including growth, stress tolerance responses and biofilm formation. In addition, the viability of the deficient mutant also suggests that SUF, the sole Fe-S cluster machinery identified is non-essential in *S. mutans*, which is not known in any other bacterium lacking the NIF and/or ISC system. However, how the bacterium compensates the Fe-S deficiency and if any novel Fe-S assembly systems exist in this bacterium await further investigation.

## Introduction

*Streptococcus mutans* is generally considered as a key etiological agent of human dental caries, a chronic infectious disease affecting billions of people worldwide (Kassebaum et al., 2015; Bowen et al., 2018). Protractive exposure to sugars, especially sucrose-rich diets facilitate *S. mutans* to accumulate on tooth surfaces. The initiation and progression of dental caries by *S. mutans* is attributed to its ability to form biofilms, to grow and metabolize carbohydrates under low pH conditions and the ability to respond rapidly to environmental stressors such as low pH, reactive oxygen species such as hydrogen peroxide, and fluctuations in the level of nutrients (Lemos et al., 2013). *S. mutans* is capable of fermenting various sugars to lactic acid and other weak acids, and produces adhesive exopolysaccharides (EPS, also glucans) from dietary sucrose and starch by the function of the glucosyltransferases (GtfBC&D) (Klein et al., 2009). The *S. mutans* derived EPS and the acidic milieu attract and promote the accumulation and coaggregation of other acidophilic and/or acidogenic microorganisms such as lactobacilli causing an ecological imbalance of the normal oral microbiota, which subsequently leads to the demineralization of the tooth enamel (Bowen et al., 2018).

Iron is an essential element for almost all living organisms and can be combined with elemental sulfur to form Fe-S clusters, which are required for various biological functions such as substrate binding/activation, transcriptional and translational regulation, iron storage and as cofactors in essential biochemical pathways (Johnson et al., 2005). These widespread roles are primarily achieved by the ability of Fe-S clusters to donate or accept electrons (Beinert and Kiley, 1999). Fe-S clusters are also used as “molecular switches” for gene regulation at both the transcriptional and translational levels due to their sensitivity to cellular redox conditions (Kiley and Beinert, 2003). Many global transcriptional regulators contain Fe-S clusters, including FNR, IscR and SoxR which were initially discovered in *Escherichia coli* but homologs have been identified in many other bacterial species (Kiley and Beinert, 2003; Rajagopalan et al., 2013). In addition, the Fe-S clusters in *E. coli* have also been shown to play a role in the catalytic activity of enzymes such as, aconitase, 6-phosphogluconate dehydratase, fumarase A, NADH dehydrogenase, and succinate dehydrogenase (Fontecave et al., 2008).

Organisms ranging from bacteria to higher eukaryotes have evolved with either one or multiple Fe-S cluster assembly pathways, including the NIF (nitrogen fixation) system, ISC (iron-sulfur cluster) system and SUF (sulfur mobilization) system (Zheng et al., 1998; Yuvaniyama et al., 2000; Takahashi and Tokumoto, 2002). The NIF system was first discovered in *Azotobacter vinelandii* with its function specific to the nitrogenase activity of the organism (Jacobson et al., 1989), but it has since been identified as a machinery for Fe-S clusters and the maturation of Fe-S proteins in many other bacteria (Fontecave and Ollagnier-de-Choudens, 2008). The ISC system was also described in *A. vinelandii* (Zheng et al., 1998), and is widely distributed among all domains of life including archaea, Gram-negative bacteria, yeasts, plants, animals and humans (Lill, 2009; Rouault, 2012). The SUF system was initially found in *E. coli*, but has since been identified in archaea, Gram-positive bacteria, cyanobacteria, plant chloroplasts and green algae (Gao, 2020). *E. coli* contains both the ISC and SUF pathways (Outten et al., 2004), with the ISC system as the housekeeping system, while the SUF system functions under harsh conditions such as oxidative stress and iron limitation (Fontecave et al., 2008). On the other hand, some Gram-positive bacteria such as *Mycobacterium tuberculosis, Bacillus subtilis* and *Staphylococcus aureus*, were shown to comprise only the SUF system (Kobayashi et al., 2003; Huet et al., 2005; Roberts et al., 2017).

Iron-sulfur (Fe-S) cluster biogenesis by the bacterial SUF pathway has been comprehensively studied in both Gram-positive and Gram-negative bacteria (Blahut et al., 2020). The Fe-S cluster biosynthesis centers around a scaffold protein or protein complex where the SufB and SufD interact directly with SufC, forming the SufBCD complex. SufC acts as an ATPase, SufD is required for Fe acquisition, and SufB is the possible site for Fe-S cluster synthesis. L-cysteine is utilized as a source of sulfur by SufS, a cysteine desulfurase that catalyzes the production of alanine and a SufS-bound persulfide through removal of elemental sulfur from cysteine. The persulfide is subsequently transferred to SufU, a sulfur transfer protein that provides the sulfur to SufBCD complex, for Fe-S cluster assembly. The synthesized Fe-S cluster is transferred directly to either an apoprotein or an Fe-S cluster carrier that traffics the cofactor to the target apoprotein.

Unlike the other systems, the SUF machinery in bacteria is fundamentally different from and does not share structural homology with the human machinery (Lill, 2009). Therefore, a therapeutic agent disrupting the SUF Fe-S synthesis machinery in bacteria is unlikely to affect essential processes of Fe-S cluster synthesis in humans, thus making it a potential candidate for targeting of the bacterial pathogens in therapeutic and preventive strategies. A small molecule inhibiting the Fe-S cluster assembly in *S. aureus* has been reported (Choby et al., 2016). Essentially, the SUF system is the only Fe-S synthesis machinery present among bacteria in the *Firmicutes* phylum, where some of the highly virulent Gram-positive bacterial pathogens are classified (Riboldi et al., 2009). Among these bacteria, the *S. aureus* SUF machinery has been adequately described (Mashruwala et al., 2015). Roberts et al. showed that knocking down *suf* transcripts decreased viability in *S. aureus*. Defective Fe-S cluster synthesis led to multiple phenotypes associated with impaired Fe-S protein maturation in the mutant (Roberts et al., 2017). The SUF system has been established to play an essential role in *M. tuberculosis’* survival against iron limitation and oxidative stress (Huet et al., 2005).

*Streptococcus mutans* does not have the ISC or NIF system, but possesses a SUF system encoded in the *sufCDSUB* cluster (Ajdic et al., 2002). The *sufCDSUB* cluster is located immediately downstream of the *uppP-mecA-rgpG* cluster (Supplementary Figure 1), which we have recently shown to play important roles in various cellular processes including cell envelope biogenesis, cell division, stress tolerance responses, and biofilm formation (Yamashita et al., 1999; De et al., 2017; De et al., 2018). The close proximity of the *uppP/mecA/rgpG* and the *sufCDSUB* clusters may suggest a potential linkage between them in functions related to cell envelope biogenesis (De et al., 2017, 2018). Here we set out to determine if *sufCDSUB* contributes to *S. mutans* cell envelope biogenesis and oxidative stress tolerance responses. The results presented here show that *sufCDSUB* is not essential for *S. mutans* viability, but its absence significantly reduces the growth rate and causes compromises in various cellular processes. Relative to the wild-type, the deficient mutant demonstrated significant growth defects especially in the absence of selected amino acids and under oxidative and nitrosative stresses. The mutant also displayed reduced survival following challenges with low pH, oxidative and nitrosative stress. The absence of *sufCDSUB* increased cell surface hydrophobicity, reduced cell wall integrity and impaired the DNA repair mechanisms. The *sufCDSUB* mutant also formed significantly reduced biofilms, a critical virulence factor for the bacterium. These results indicate that *sufCDSUB* cluster influences major phenotypic characteristics that affects the pathophysiology of *S. mutans.*

## Materials and Methods

### Bacterial Strains and Culture Conditions

*Streptococcus mutans* strain UA159 and its derivatives (Table 1) were maintained in brain heart infusion (BHI; Difco Laboratories, Detroit, MI) with and without the addition of antibiotics kanamycin (Kan, 1 mg/mL), erythromycin (Erm, 10 μg/mL), and/or spectinomycin (Spc, 1 mg/mL), when necessary. Solid media were prepared by adding 1.5% (w/v) Bacto agar (Difco Laboratories, Detroit, MI, United States). Unless otherwise stated, cultures were maintained in an aerobic chamber at 37^*o*^C with inclusion of 5% CO_2_. To study the growth of the bacterial strains in the absence of particular amino acids, chemically defined medium FMC with glucose as the carbohydrate source (Terleckyj et al., 1975; Wen et al., 2017) was used with slight modifications. *E. coli* strains were grown in LB with and without inclusion of antibiotic Spc (100 μg/mL) and Kan (40 μg/mL).

**TABLE 1:** Bacterial strains and plasmids used in this study.

Strains/Plasmid	Major genotypes and phenotypes	References
*S. mutans* UA159	wild-type (ATCC 700610)	ATCC
*S. mutans* TW424	Δ*sufCDSUB::kan*^*r*^, *sufCDSUB* deleted and replaced with *kan*^*r*^, Kan^r^	This study
*S. mutans* TW464	Δ*sufCDSUB::erm*^*r*^, *sufCDSUB* deleted and replaced with *erm*^*r*^, Erm^r^	This study
*S. mutans* TW498	Δ*sufB::kan*^*r*^, *sufB* deleted and replaced with *kan*^*r*^, Kan^r^	This study
*S. mutans* TW503	Δ*sufC::spc*^*r*^, *sufC* deleted and replaced with *spc*^*r*^, Spc^r^	This study
*S. mutans* TW499	*sufC::kan*^*r*^, inactivation of *sufC* via insertion of *kan*^*r*^, Kan^r^	This study
*S. mutans* TW500	Δ*sufD::kan*^*r*^, *sufD* deleted and replaced with *kan*^*r*^, Kan^r^	This study
*S. mutans* TW501	Δ*sufU::erm*^*r*^, *sufU* deleted and replaced with *erm*^*r*^, Erm^r^	This study
*S. mutans* TW502	Δ*sufS::kan*^*r*^, *sufS* deleted and replaced with *kan*^*r*^, Kan^r^	This study
*S. mutans* TW476	Δ*sufCDSUB::*erm*/sufCDSUB* plus its cognate promoter, Kan^r^, Erm^r^	This study
*E. coli* DH10B	Cloning host, *mcrA*, *mcrBC*, *mrr*, and *hsd*	Invitrogen, Inc.
pBGK3	Integration vector, Kan^r^	De et al., 2017
pFW11	Integration vector with a promoterless luciferase reporter, Spc^r^	Kreth et al., 2005
pFWP*suf*	pFW11 fused with *suf* promoter, Spc^r^	This study
pFW*PyrA*	pFW11 fused with *pyrA* promoter, Spc^r^	This study
pFWP*ldh*	pFW11 fused with *ldh* promoter, Spc^r^	Bitoun et al., 2012

*kan^*r*^, erm^*r*^ and spc^*r*^ for non-polar kanamycin resistance (Kan^*r*^), non-polar erythromycin resistance (Erm^*r*^), and non-polar spectinomycin resistance (Spc^*r*^), respectively.*

### Genetic Manipulations

The mutants were constructed by allelic exchange mutagenesis with the deletion and replacement of the *sufCDSUB* gene cluster as a whole or individually by a non-polar kanamycin, spectinomycin or erythromycin resistance element as similarly described previously (Lau et al., 2002; Zeng et al., 2006; Bitoun et al., 2013). Briefly, the 5′ and 3′ flanking regions with the size around 1.0 kb of the target gene(s) were PCR amplified with proper restriction sites incorporated using gene-specific primers (Supplementary Table 1) and high fidelity DNA polymerase Q5 (New England Biolabs). Following Sanger sequencing to verify sequence accuracy, the PCR amplicons were digested with proper restriction enzymes and then ligated to a non-polar antibiotic resistance element that was digested with similar enzymes. The ligation mixture was then used to directly transform *S. mutans* with the inclusion of competence stimulating peptide (CSP) (Li et al., 2001), and mutants deficient of target gene(s) were selected on BHI agar plates supplemented with proper antibiotics. The selected mutants were further examined by PCR analysis using the far-end 5′ and 3′ primers (Supplementary Table 1) and by Sanger sequencing to verify the accuracy of the allelic exchange mutagenesis. For complementation, the *sufCDSUB* cluster along with its putative promoter was amplified by high fidelity Q5 DNA polymerase, and cloned into integration vector pBGK3 (Wen and Burne, 2001; De et al., 2017). Following sequence verification by Sanger sequencing, the resulting construct was used to transform the *sufCDSUB* mutant, and the complement strain as a result of double crossover homologous recombination was isolated from BHI plates with Erm and Kan. For transcriptional analysis of *sufCDSUB*, the putative promoter region of the *sufCDSUB* cluster was cloned in front of the promoterless luciferase gene in pFW11 (Kreth et al., 2005), and the resulting plasmid was transformed into *S. mutans* wild-type UA159 by following established procedures (De et al., 2018).

### Growth Analyses

*Streptococcus mutans* strains were cultured overnight and diluted 1:100 in different media with a mineral oil overlay and the optical density of the cultures at 600 nm was monitored continuously at 37°C using a Bioscreen C (Bitoun et al., 2013). The growth in response to different pH was tested in BHI medium adjusted to pH 6.0. Growth under oxidative stress and nitrosative stress was assessed using BHI supplemented with 12.5 mM and 25 mM methyl viologen and with 2 mM sodium nitroprusside (Sigma-Aldrich), respectively. To assess the amino acid requirement for growth, the chemically defined FMC medium was used (Terleckyj et al., 1975), with omission of selected amino acids whose biosynthesis are known to require the Fe-S cluster including isoleucine, leucine, or glutamic acid and glutamine as described previously with slight modifications (Roberts et al., 2017). The growth rate in doubling time was calculated using standard methods from three separate experiments (Bitoun et al., 2013).

### Acid, Oxidative and Nitrosative Stress Tolerance Assays

The effect of *sufCDSUB*-absence on the ability of *S. mutans* to withstand acid and oxidative stresses were assessed using acid killing and hydrogen peroxide challenge assays as described previously (Wen and Burne, 2004). Briefly, planktonic cultures of the *S. mutans* strains were grown in BHI until mid-exponential phase (OD_600 *nm*_ = 0.3–0.4) and then subjected to acid killing by incubating in 0.1 M glycine buffer, pH 2.8 and hydrogen peroxide killing in 0.1 M glycine buffer containing hydrogen peroxide at 0.3% (w/v, or 58 mM final concentration) for periods of times specified (Wen and Burne, 2004). For adaptive acid tolerance response, the bacterial cultures were harvested by centrifugation at 3,000 × *g* at 4°C for 5 min and the cell pellets were resuspended and incubated in BHI adjusted to pH 5.0 for one hour before subjected to acid killing assay as described above. The ability to withstand nitrosative stress was analyzed by exposing the bacterial cells to 50 mM sodium nitroprusside in 0.1 M glycine buffer and the number of cells survived was determined at the time intervals specified (Roberts et al., 2017).

### Glycolytic pH Drop

The glycolytic pH drop assay was carried out as described by Belli and Marquis (1991). Briefly, *S. mutans* strains were grown in BHI broth until mid-exponential phase (OD_600 nm_ ≅ 0.4), washed twice with ice-cold de-ionized water by centrifugation at 3000 × *g* at 4°C for 10 min, and the cells were then resuspended in 50 mM KCl and 1 mM MgCl_2_. The pH was adjusted to 7.2 with 0.1 M KOH before the addition of 50 mM glucose. The pH drop was monitored continuously for a period of 20 min.

### Biofilm Analysis

For biofilm formation, *S. mutans* UA159 strains were cultured in modified semi-defined biofilm medium (BM) with glucose (20 mM, BMG), sucrose (20 mM, BMS), and/or glucose plus sucrose (18 mM and 2 mM, respectively, BMGS) as the supplemental carbohydrate source (Loo et al., 2000; Wen and Burne, 2002). Biofilms were grown on polystyrene 96-well plates (Corning, New York) or hydroxylapatite disks (HA) as previously described (Wen and Burne, 2002, 2004). For the 96-well plate model, biofilms were stained using 0.1% crystal violet and subsequently quantitated using a spectrophotometer at 575 nm (Wen and Burne, 2002). For imaging the biofilms, hydroxylapatite (HA) disks vertically deposited in 24 well plates (Corning, NY, United States) were incubated with bacterial cultures for 24 h, and analyzed using confocal laser scanning microscopy (CLSM) as described previously (Wen et al., 2006; Bitoun et al., 2013). Post-acquisition, the biofilm biovolume and average biofilm thickness of the biofilms were quantitated using COMSTAT (Heydorn et al., 2000; De et al., 2018).

### Determination of pH Profiles and Lactic Acid Concentrations

The end-point pH of the filtered spent culture medium of the *S. mutans* strains was measured using a pH electrode (Fisher Scientific Accumet AB15 pH mV meter). The concentration of lactic acid in the spent medium was determined using an EnzyChrom^TM^ L-Lactate Assay Kit (ECLC-100) (BioAssay Systems, CA, United States) according to the manufacturer’s instructions.

### Congo Red Sensitivity Assay

Overnight cultures of the *S. mutans* UA159 and Δ*suf::kan^*r*^* mutant were transferred to fresh BHI medium and grown until OD 0.5 at 600 nm. Then the cultures were serially diluted and 5 μL of each dilution were spotted on BHI agar plates and simultaneously on BHI agar plates containing 0.2% (w/v) Congo Red (Sigma-Aldrich). The plates were incubated for 24 h, and growth in number and size of the colonies were examined.

### Total Exoprotein Analyses

Spent medium supernatants of the *S. mutans* UA159 and Δ*suf::kan^*r*^* were obtained from their respective overnight biofilm cultures by centrifugation at 3000 × *g* at 4°C for 10 min, and the culture medium was filter sterilized with a 0.22 μm syringe filter (Millex). Exoproteins in the spent medium were extracted using 10% (w/v) trichloroacetic acid precipitation. The resultant protein pellets were resuspended in a buffer containing 60 mM Tris, pH 6.8, 10% glycerol and 5% SDS. Protein concentrations were determined using the Bicinchoninic acid (BCA) assay (Pierce). The data were further normalized to the number of colony-forming-units in the overnight culture.

### Extracellular Deoxyribonucleic Acid (eDNA) Analyses

Overnight cultures of *S. mutans* UA159 and Δ*suf::kan^*r*^* were transferred into 50 mL tubes with a sterile glass slide containing BM medium supplemented with 18 mM glucose and 2 mM sucrose and incubated overnight at 37°C in a 5% CO_2_ incubator. After incubation, the biofilms on the surfaces of the glass slides were harvested by scratching off with a pipette and washed with phosphate buffered saline (PBS), pH 7.5. Following brief sonication (Liao et al., 2014), the biofilm cells were pelleted by centrifugation at 3000 × *g* for 10 min at 4°C and the supernatants were collected and the eDNA was precipitated by ethanol precipitation with 2 volumes of absolute ethanol, 1/10 volume of 3 M sodium acetate, pH 5.2 and 1 mM EDTA (final concentration) at −80°C overnight. The precipitated eDNA was centrifuged at 21,130 × *g* at 4°C for 20 min and the pellets were washed with 70% ice cold ethanol and air dried. The eDNA was resuspended in 400 μL of sterile DNase/RNase free water and RNase A treated with 100 μg/mL (final concentration) at 37°C for 1 h. Subsequently, the DNA concentrations were measured using a Nanodrop 2000 spectrophotometer (Thermo scientific), and the results were further normalized with colony-forming units.

### Mutagenesis Frequency

*Streptococcus mutans* UA159 and Δ*suf::kan^*r*^* cultures were grown overnight and diluted 1:100 in fresh BHI medium supplemented with rifampicin (Sigma-Aldrich) at a sub-minimum inhibitory concentration of 0.015 μg/mL (ZT Wen, personal communication). The cells were cultured for 48 h at 37°C, aerobically in the presence of 5% CO_2_ with shaking to induce the growth of cells resistant to rifampicin. Cultures were then serially diluted and plated on BHI agar to determine the total CFU and also spread plated on BHI agar supplemented with 0.03 μg/mL of rifampin to enumerate the number of mutants resistant to rifampicin. The mutagenesis frequency was calculated by dividing the number of rifampicin resistant colonies by the total number of colonies.

### Reverse Transcription-Polymerase Chain Reaction

Actively growing mid-exponential phase cultures of the wild-type *S. mutans* were used for RNA extraction (Wen et al., 2006). Briefly, the bacterial cells were harvested by centrifugation at 4°C for 5 min, and then immediately treated with RNA Protect^TM^ (Qiagen, Inc.). Total RNA extraction was performed using a hot phenol method (Wen and Burne, 2004; Wen et al., 2006). Extracted RNAs were DNase I treated (Ambion) and subsequently cleaned up using the RNeasy purification kit (Qiagen, Inc.). Reverse transcription and cDNA synthesis were performed using SuperScript^TM^ III First-Strand Synthesis System (ThermoFisher Scientific), using gene specific primers (Supplementary Table 1) similarly as described (Wen et al., 2006). To check the co-transcription of the *sufCDSUB* gene cluster, the resulting cDNA was PCR amplified using gene-specific forward and reverse primers (Supplementary Table 1), designed to amplify regions starting ∼50 bp upstream and ∼100 bp into the coding region of each gene of the *sufCDSUB* cluster. Genomic DNA (gDNA) was used as the positive control and reactions where the reverse transcriptase enzyme was not included during the RT reaction were used as the negative control.

### Luciferase Reporter Assays

Luciferase reporter assays were performed as previously described (Podbielski et al., 1999; Kreth et al., 2005; Bitoun et al., 2012). Briefly, the reporter strains cultured overnight in BHI medium with proper antibiotics were diluted 1:100 in fresh BHI, BHI with pH 6.5 and BHI with inclusion of 2.5 mM Methyl Viologen. Aliquots of 200 μL bacterial cultures were collected at early- (OD_600_ ∼0.2), mid- (OD_600_ ∼0.4) and late- (OD_600_ ∼0.6) exponential phase of growth and added into a 96 well plate (CELLSTAR). Luciferase activity was assessed by adding 25 μL of 1 mM D-luciferin (Gold Biotechnology). For control, pFW11 with promoterless reporter and a luciferase reporter fused with the constitutive *pyrA* promoter were used. In some cases, the luciferase reporter fused to the promoter of the constitutive *ldh* gene was also used. The relative light units were normalized to the OD_600 *nm*_ at each time-point.

### Cell Surface Hydrophobicity Assay

Cell surface hydrophobicity was measured using the method of Rosenberg with minor modifications (Rosenberg et al., 1980). Briefly, *S. mutans* strains were grown in BHI broth until mid-exponential phase (OD_600 *nm*_ ∼0.4), harvested by centrifugation at 4°C at 3000 × *g* for 5 min, and washed once with PBS. The cell pellets were resuspended in PUM (phosphate, urea, magnesium) buffer to OD ∼0.6. Aliquots of 3.5 mL of the cell suspension was overlaid by 0.6 mL decane (MillporeSigma, St. Louis, MO, United States). After vigorous votexing, phases were allowed to separate for 15 min at room temperature, and the OD_400 *nm*_ of the aqueous phase was measured and recorded before (A_0_) and after mixing (A_1_). The percentage of hydrophobicity was calculated as follows: Hydrophobicity (%) = [(A_0_−A_1_)/A_0_]×100.

### Statistical Analysis

Unless otherwise stated, all experiments were performed in three separate times. Quantitative data were analyzed using the Student *t*-test. A *P*-value of 0.05 or less is considered statistically significant.

## Results

### Absence of *sufCDSUB* Causes Significant Growth Defects in the Deficient Mutants

**FIGURE 1:**
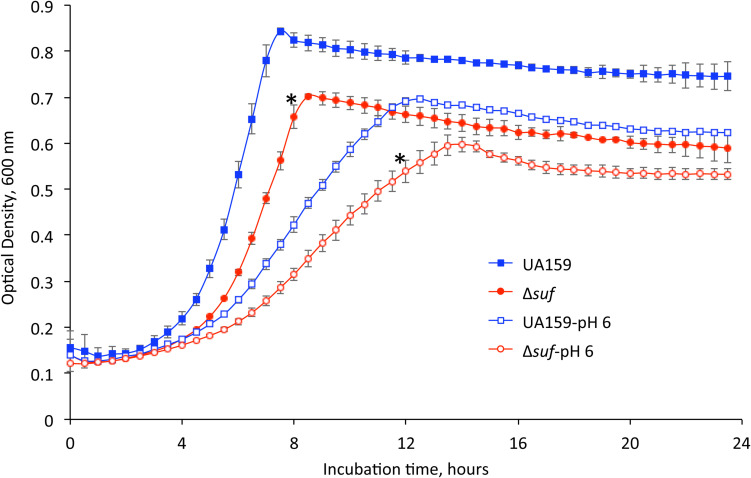
Growth characteristics of the mutant strains. *Streptococcus mutans* wild-type, UA159 and the *sufCDSUB-*deficient strain, Δ*suf::kan^*r*^* (Δ*suf*) were grown in BHI broth and BHI broth with pH adjusted to 6.0. The optical densities of the cultures at 600 nm were recorded continuously using a Bioscreen C. Data presented here are representatives of three separate experiments with symbol * indicating a statistically significant difference in the doubling time between UA159 and Δ*suf::kan^*r*^* under the same conditions with a *P* < 0.05.

In comparison to the wild-type, UA159, the allelic exchange mutant with the *sufCDSUB*-deleted and replaced with a non-polar kanamycin resistance marker (*kan*^*r*^), Δ*suf::kan^*r*^*, showed significant defects in growth (Figure 1). As compared to UA159, Δ*suf::kan^*r*^* had a significantly lower growth rate with an average doubling time of 2.83 (± 0.25) hours vs 1.81 (± 0.05) hours for UA159 (*P* < 0.05). Similar results were also obtained with Δ*suf::erm^*r*^* which has the *sufCDSUB-*coding regions replaced with a non-polar erythromycin resistance element (*erm*^*r*^) (Supplementary Figure 2A). Complementation of the Δ*suf::erm^*r*^* mutant in strain Δ*suf*^+^ with a copy of the wild-type *sufCDSUB* cluster plus its cognate promoter region restored the phenotype to that of the wild type (Supplementary Figure 2A). Allelic exchange mutants Δ*sufC::spc^*r*^*, Δ*sufD::kan^*r*^*, Δ*sufS::kan^*r*^*, Δ*sufU::erm^*r*^*, and Δ*sufB::kan^*r*^*, which have the respective gene *sufC, -D, -S, -U*, and *-B* deleted individually using similar strategies as described above, did not show any significant effects on growth as the Δ*suf::kan^*r*^* and Δ*suf::erm^*r*^*, compared to their parent strain (Supplementary Figure 2A and Supplementary Table 2). Neither did the *sufC::kan^*r*^* mutant that has *sufC* disrupted by insertion of the non-polar kanamycin resistance marker (Supplementary Figure 2A). In agreement with the reduction in the growth rate, the final pH of the spent medium of mutant Δ*suf::kan^*r*^* was also higher than the parent strain when grown in regular BHI broth (Supplementary Figure 3).

As shown in Figure 1 and Supplementary Figure 2B during growth in BHI adjusted to pH 6.0, the *suf* mutant, Δ*suf::kan^*r*^* also showed an extended lag phase followed by a longer doubling time of 4.68 (± 0.15) hours (*P* < 0.05), as compared to the wild-type, UA159 with an average of 3.96 (± 0.75) hours and the complement strain, Δ*suf*^+^ which had a doubling time of 3.92 (± 0.22) hours. Similarly, the *sufB*-deletional mutant, Δ*sufB::kan^*r*^*, also showed an extended lag phase and a lower growth rate, although none of the other mutants with single gene mutation showed any major difference when compared to the wild-type (Supplementary Figure 2B and Supplementary Table 2).

### Deficiency of *sufCDSUB* Affects the Biosynthesis of Specific Amino Acids

**FIGURE 2:**
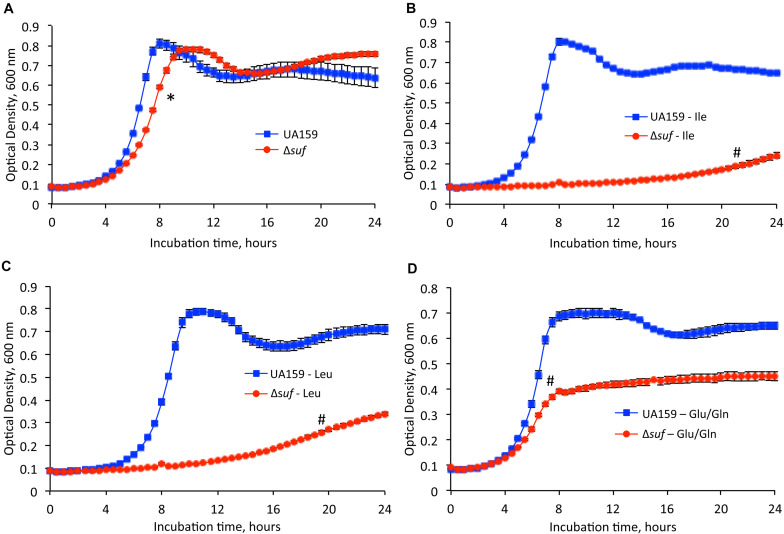
Growth studies in the absence of selected amino acids. *Streptococcus mutans* wild-type, UA159 and the *sufCDSUB-*deficient mutant, Δ*suf::kan^*r*^* (Δ*suf*) were grown in **(A)** FMC medium, **(B)** FMC medium with omission of isoleucine, **(C)** FMC medium with omission of leucine, **(D)** FMC medium with omission of glutamic acid (Glu) and glutamine (Gln). Data presented are representatives of three separate experiments. Symbol * and # indicates a statistical significance at *P* < 0.05 and *P* < 0.001, respectively, when compared to UA159 under the same conditions tested.

To test whether *S. mutans* require functional Fe-S proteins for the synthesis of some essential amino acids, the growth of the wild-type, UA159 and Δ*suf::kan^*r*^* mutant were tested using chemically defined medium FMC in the presence and absence of isoleucine, leucine, or glutamate/glutamine, amino acids whose biosynthesis requires the functional Fe-S cluster requiring enzymes (Figure 2 and Supplementary Table 2). Similarly, when grown in FMC with all the essential amino acids included, Δ*suf::kan^*r*^* showed a reduced growth rate, as compared to the wild-type (Figure 2A; *P* < 0.05). However, when grown in medium without isoleucine (Figure 2B) and leucine (Figure 2C), Δ*suf::kan^*r*^* demonstrated a dramatically extended lag phase and a significantly longer doubling time than the parent strain (Supplementary Table 2; *P* < 0.001). While UA159 demonstrated a maximum culture density of OD_600_ of 0.793 (± 0.005) and 0.789 (± 0.013) in the absence of isoleucine and leucine, respectively, Δ*suf::kan^*r*^* had a maximum culture density OD_600_ of 0.241 (± 0.015) and OD_600_ 0.338 (± 0.007), respectively (*P* < 0.001). In the absence of glutamate/glutamine (Figure 2D), Δ*suf::kan^*r*^* had a longer doubling time and a reduced culture density, when compared to UA159 (*P* < 0.001 for both).

### Deletion of *sufCDSUB* Led to Weakened Glycolytic pH Drop and Acid Tolerance Response

**FIGURE 3:**
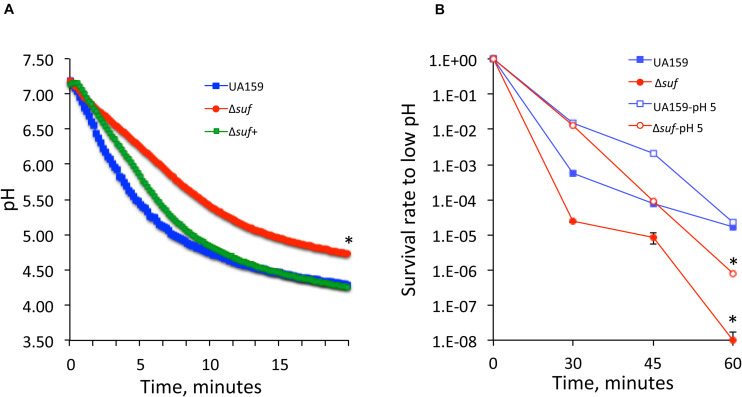
Glycolytic pH drop analysis and acid killing assays. **(A)** Results of pH drop experiments showed that relative to wild-type UA159, the Δ*suf::kan^*r*^* mutant (Δ*suf*), had a significantly slower pH drop and a higher resting pH after 20 min (*, *P* < 0.05). Complementation with wild-type *sufCDSUB* in Δ*suf*^+^ was able to restore the phenotype to UA159. **(B)** When subjected to acid killing by incubation in buffer of pH 2.8, the survival rate of Δ*suf::kan^*r*^* was reduced by 2-logs than wild-type (UA159) after 30 min and became virtually undetectable after 60 min. Although like UA159, it was able to increase its survival rate following pre-incubation at pH 5.0 for one hour. *, *P* < 0.001.

The ability to survive and adapt harsh environment such as the low pH and oxidative stressors is known as a trait critical to *S. mutans’* cariogenicity (Burne, 1998). Reduction of growth rate during growth in BHI adjusted to lower pH indicates a reduced glycolytic activity and/or weakened acid tolerance. When analyzed using glycolytic pH drop experiments, it was found that compared to UA159, the rate (slope) of glucose-induced pH drop of the *suf* deficient mutant, Δ*suf::kan^*r*^* was significantly reduced and the resulting pH of the *suf* mutant was significantly higher (*P* < 0.05) (Figure 3A). As expected, complementation of the mutant restored the phenotype similar to the wild-type. The effect of the absence of *sufCDSUB* on the ability of *S. mutans* to withstand low pH was further assessed using the acid killing by incubation in a buffer of pH 2.8 (Wen and Burne, 2004). The results showed that relative to the parent strain, the survival rate of the Δ*suf::kan^*r*^* mutant decreased by ∼2-logs after 30 min and > 3-logs (virtually undetectable) after 60 min (Figure 3B; *P* < 0.001).

*Streptococcus mutans* is known to possess an adaptive acid tolerance response which is featured with enhanced acid tolerance following initial exposure to low pH environment (Lemos and Burne, 2008). To examine if the absence of *sufCDSUB* affects the constitutive acid tolerance and/or adaptive acid tolerance response, *S. mutans* strains were incubated in BHI adjusted to pH 5.0 for one hour before subjecting to acid killing assays as described above. As shown in Figure 3B, the results showed that while the differences were not as dramatic as the non-adapted cells, significant reduction in survival rate was measured consistently with the Δ*suf::kan^*r*^* mutant. However, relative to the non-adapted strains, both the Δ*suf::kan^*r*^* mutant and its parent strain displayed significant increases in their survival rates following initial incubation in BHI of pH 5.0 (*P* < 0.001). These results further suggest that the absence of *sufCDSUB* weakens acid tolerance responses and mostly to the constitutive acid tolerance response rather than the adaptive acid tolerance response.

### Deletion of *sufCDSUB* Resulted in Increased Susceptibility of the Deficient Mutants to Oxidative and Nitrosative Stressors

**FIGURE 4:**
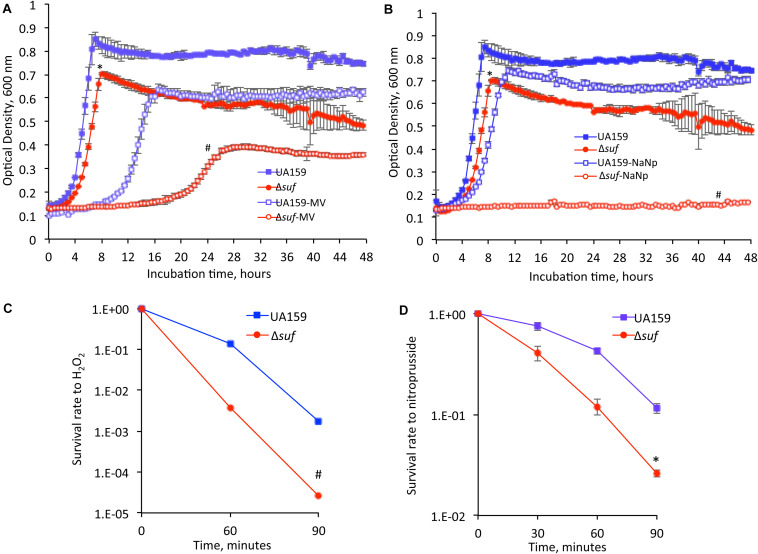
Oxidative and nitrosative stress challenging assays. When grown in the presence of methyl viologen (MV) at 25 mM **(A)** and sodium nitroprusside (NaNp) at 2 mM **(B)**, the Δ*suf::kan^*r*^* mutant (Δ*suf*), showed significantly reduced growth rate and especially, the culture density as measured using a Bioscreen C, as compared to the wild-type. Symbol * and # indicates statistical significance at *P* < 0.05 and < 0.001, respectively. Following incubation in the presence of hydrogen peroxide (H_2_O_2_) at 58 mM **(C)** or sodium nitroprusside at 50 mM **(D)**, the Δ*suf* mutant showed 2-log and 2-fold reduction, respectively, when compared to the wild-type. Symbol * and # indicates significant differences at *P* < 0.001 and < 0.05, respectively.

The ability of the Δ*suf::kan^*r*^* mutant to adapt to oxidative stress conditions was first tested by supplementing BHI medium with methyl viologen at 12.5 mM and 25 mM (final concentration), which produces superoxide radicals and hydrogen peroxide (Wen et al., 2011). At 12.5 mM, Δ*suf::kan^*r*^* and Δ*suf::erm^*r*^* grew at similar growth rate but had a significantly reduced culture density compared to the wild-type (Supplementary Figure 4; *P* < 0.05). None of the mutants with single gene deletion/inactivation showed any major difference in growth from the parent strain. However, when grown in BHI with methyl viologen at 25 mM, Δ*suf::kan^*r*^* displayed an extended lag phase of > 20 h and a dramatic reduction in culture density after 48 h, as compared to the wild-type (Figure 4A; *P* < 0.001).

Similarly, the *suf* deficient mutants were tested for their ability to grow under nitrosative stress conditions by supplementing BHI with sodium nitroprusside (2 mM, final concentration), which breaks down inside the cells through interactions with sulfhydryl-containing compounds such as cysteine and glutathione to yield nitric oxide, a major reactive nitrogen species. In comparison to the wild-type, the Δ*suf::kan^*r*^* mutant displayed an extended lag phase and yielded only limited growth with a culture density of ∼5-folds less compared to the wild-type, after 48 h (*P* < 0.001) (Figure 4B and Supplementary Figure 5). The complement strain, Δ*suf*^+^ showed a phenotype similar to the wild-type. The mutants with individual gene deleted also displayed a significant reduction in growth with extended lag phases in the presence of sodium nitroprusside, compared to the wild type (Supplementary Figure 5; *P* < 0.001).

The mutant was further analyzed by H_2_O_2_ challenge assay by incubation in a buffer containing H_2_O_2_ at 58 mM (final concentration), and the results showed that relative to the wild-type, the survival rate of the Δ*suf::kan^*r*^* mutant decreased by 2-logs after 60 min (Figure 4C; *P* < 0.001). To determine the ability of the wild-type and Δ*suf::kan^*r*^* mutant to withstand nitrosative stresses, the bacterial cells were exposed to a buffer containing sodium nitroprusside (50 mM, final conc.), and a reduction in survival rate by ∼2-fold was also observed with mutant Δ*suf::kan^*r*^*, as compared to the wild type after 90 minutes of exposure (Figure 4D; *P* < 0.05).

### Absence of *sufCDSUB* Leads to Compromises in Biofilm Formation

When grown in BM broth medium with glucose (BMG), the Δ*suf::kan^*r*^* mutant had the tendency to form small aggregates settling in the bottom of the test tubes, as compared to the wild-type, UA159 (Supplementary Figure 6). Similar phenomenon was also observed when grown in BM with glucose and sucrose (BMGS), but less so in BM with sucrose alone (BMS). When the pH of the overnight culture medium was evaluated, both Δ*suf::kan^*r*^* and Δ*suf::erm^*r*^* mutants had a higher pH than the wild-type, UA159 with the highest difference observed when grown in BMS (Supplementary Figure 7A). There were no significant differences in the lactate concentration between the wild-type and the *suf* mutants, Δ*suf::kan^*r*^* and Δ*suf::erm^*r*^* (Supplementary Figure 7B).

**FIGURE 5:**
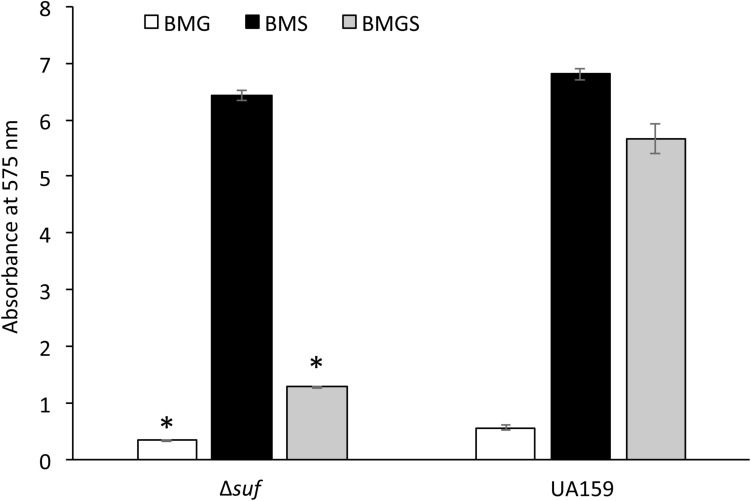
Analysis of biofilm formation on 96-well plates. *Streptococcus mutans* wildtype, UA159 and the Δ*suf::kan^*r*^* mutant (Δ*suf*) were grown in BM medium with glucose (BMG), sucrose (BMS), and glucose plus sucrose (BMGS), and biofilms on the polystyrene plates were analyzed using a spectrophotometer. Data represented here are means (± standard deviation) of at least three independent experiments, with ^∗^ indicating significant differences at *P* < 0.05.

When grown in the 96-well plates, the wild-type formed substantial biofilms especially during growth in sucrose-containing medium BMS and BMGS (Figure 5). In comparison, biofilm formation by mutant Δ*suf::kan^*r*^* was reduced by > 2-fold when it was grown in BMGS after 24 h (*P* < 0.05) compared to the wild-type, UA159. Similar reductions were also observed with biofilms grown in BMG, although such differences were often negated as the biofilms on BMG were loose and often got washed away during the processes. Interestingly, no significant differences between the wild-type and the Δ*suf::kan^*r*^* mutant biofilms were measured when grown in BM-sucrose alone. Similarly, Δ*suf::erm^*r*^*, Δ*sufC::spc^*r*^*, *sufC::kan^*r*^*, Δ*sufD::kan^*r*^*, Δ*sufU::erm^*r*^*, Δ*sufS::kan^*r*^*, and Δ*sufB::kan^*r*^* also formed significantly less biofilms than the wild-type when grown in BMGS medium, but not in BMS (Supplementary Figure 8A). As expected, the complement strain, Δ*suf*^+^, formed biofilms similarly to the wild-type (Supplementary Figure 8B). Some significant reductions were also measured when the incubations were extended to 48 h without medium refreshment, although the results were not consistent. It awaits further investigation how the absence of *suf* affects biofilm persistence.

**FIGURE 6:**
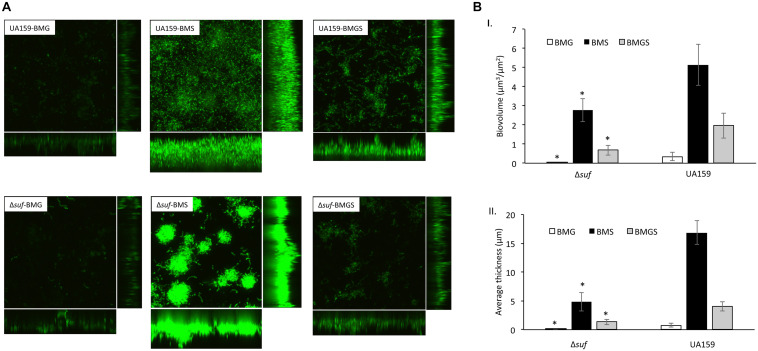
Confocal microscopic and COMSTAT analysis of biofilms. *Streptococcus mutans* wild-type UA159 and Δ*suf::kan^*r*^* mutant (Δ*suf*) were grown on hydroxylapatite disks in BM medium with glucose (BMG), sucrose (BMS), and glucose plus sucrose (BMGS), and the 24 h biofilms were analyzed using confocal microscopy. Following image acquisition, quantitation of the biofilm biovolume and average thickness were further analyzed using COMSTAT. Panel **A** shows representative restructured three-dimensional images of the biofilms of the wild-type and mutant Δ*suf::kan^*r*^*. Panel **B** shows results of the COMSTAT analysis of biovolume (I) and thickness (II). The data presented here are means (± standard deviation) of more than three independent experiments, with * indicating significant differences at *P* < 0.05.

When analyzed using confocal microscopy with biofilms stained with *Live/Dead Bac*Light staining kit (Invitrogen), biofilms grown on HA disks by the *sufCDSUB* deficient mutant, Δ*suf::kan^*r*^* demonstrated significant differences in both its architecture and quantity as compared to the wild-type (Figures 6A,B). Unlike the parent strain that formed thick and evenly distributed biofilms in the BMS medium, Δ*suf::kan^*r*^* mutant formed biofilms that existed in patches of large aggregates, especially during growth in BMS and to a lesser degree in BMGS (Figure 6A). Post-acquisition analysis via COMSTAT showed that compared to the wild-type, UA159, mutant Δ*suf::kan^*r*^* had a significantly reduced biovolume and thickness especially when grown in BMGS and BMS (*P* < 0.05) (Figure 6B). Relative to UA159, the biovolume of Δ*suf::kan^*r*^* biofilms reduced by 2.87-fold with an average of 0.68 (± 0.26) μm^3^/μm^2^ vs 1.95(± 0.84) μm^3^/μm^2^ for UA159 when grown in BMGS medium (Figure 6B-I). Similar reductions were also measured when grown in BMS with the mutant averaging 2.78(± 0.61) μm^3^/μm^2^ vs 5.13(± 1.06 μm^3^/μm^2^) for UA159 (*P* < 0.05). Δ*suf::kan^*r*^* showed > 3.5-fold reduction in biofilm thickness with an average of 4.79(± 2.09) μm during growth in BMS compared to UA159 averaging 16.86(± 1.59) μm (Figure 6B-II; *P* < 0.05). However, no major differences in live/dead ratio were observed between the Δ*suf::kan^*r*^* mutant and its parent strain (Figure 6A).

### Absence of *sufCDSUB* Results in Elevated Release of eDNA and Extracellular Proteins

**FIGURE 7:**
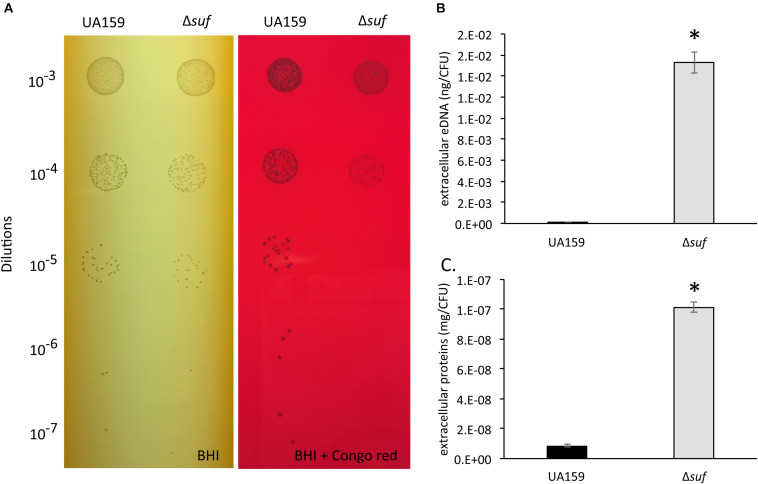
Congo Red sensitivity assay, extracellular DNA and extracellular protein analysis. **(A)** Resistance to Congo Red has been attributed to the integrity of the cell wall. The growth of *Streptococcus mutans* wild-type, UA159 and the Δ*suf::kan^*r*^* mutant (Δ*suf*) was evaluated on agar plates containing Congo Red with Δ*suf::kan^*r*^* showing reduced resistance when compared to its parent strain, UA159. The extracellular DNA concentrations **(B)** and the extracellular protein concentrations **(C)** of the wild-type and Δ*suf::kan^*r*^* were measured. The values were further normalized using the number of colony-forming units (CFU), and the data represent more than two independent sets of experiments of three replicates each, with * indicating a statistically significant difference at *P* < 0.05.

Resistance to Congo Red, a cell wall-disrupting benzidine-type dye, has been used as an indication of the integrity of the cell wall (Suzuki et al., 2012). The ability of the *S. mutans* wild-type and the Δ*suf::kan^*r*^* mutant to grow in the presence of Congo Red was analyzed by spotting serial dilutions of the bacterial cultures onto BHI agar plates with or without supplementation of 0.2% (w/v) Congo Red. In comparison to the wild-type, Δ*suf::kan^*r*^* mutant exhibited an increased sensitivity to Congo Red (Figure 7A). It is known that weakened cell envelope could lead to increases in the release of extracellular deoxyribonucleic acids (eDNA) in *S. mutans* and other bacteria (Nilsson et al., 2019). When the eDNA released in the biofilms was extracted and measured, the cell-free culture medium of the mutant was found to possess as much as 102-fold higher amount of eDNA than the wild-type (Figure 7B; *P* < 0.001). Similarly, the Δ*suf::kan^*r*^* mutant also had an 11-fold increases in the amount of extracellular protein content in the cell free culture supernatant as estimated by BCA assay (Figure 7C; *P* < 0.001) and by SDS–PAGE (Supplementary Figure 9), when compared to the wild type. These results further suggest that deletion of *sufCDSUB* in *S. mutans* may have led to compromises in the cell envelope stability.

### Deletion of *sufCDSUB* Led to an Increased Spontaneous Mutation Rate

Iron-sulfur clusters are known to be necessary for the function of DNA repair enzymes, a key component in the prevention of mutations in *rpoB*, which encodes the beta subunit of RNA polymerase (Roberts et al., 2017). Rifampicin prevents transcription initiation by binding to the beta subunit of RNA polymerase. Mutations in *rpoB* can change the spatial form of the RNA polymerase enzyme resulting in development of rifampicin resistance (Ezekiel and Hutchins, 1968; Tavanaee Sani et al., 2018). In comparison to the wild-type, the Δ*suf::kan^*r*^* mutant showed ∼1.80 (± 0.16)-fold increase in the number of rifampicin resistant cells when grown in medium with sub-minimum concentration of rifampicin (*P* < 0.05). These results can be in part attributed to inactivation/malfunction of the Fe-S cluster requiring DNA repair enzymes in response to the absence of *sufCDSUB*, which has been shown to play a role in preventing *rpoB* mutation (Porello et al., 1998; Yeeles et al., 2009).

### Deletion of *sufCDSUB* Caused an Increased Cell Surface Hydrophobicity

**FIGURE 8:**
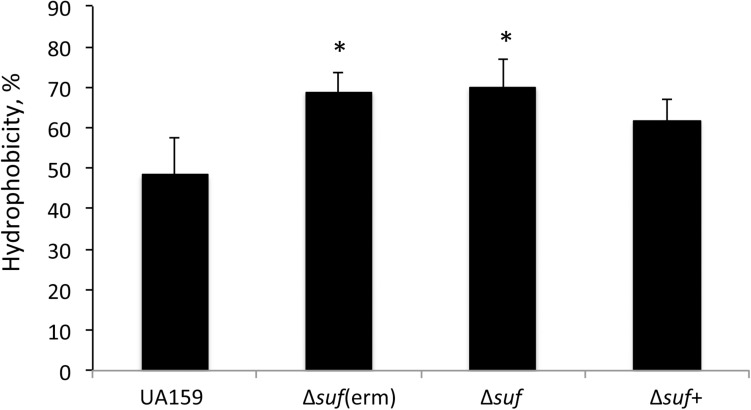
Cell surface hydrophobicity analysis. *Streptococcus. mutans* wild-type UA159, its Δ*suf::erm^*r*^* [Δ*suf*(erm)] and Δ*suf::kan^*r*^* (Δ*suf*), and the complement strain Δ*suf* + were grown in BHI broth until mid-exponential phase (OD_600 *nm*_ ∼0.4), harvested by centrifugation, and washed once with phosphate buffered saline. The cell pellets were resuspended in PUM (phosphate, urea, magnesium) buffer and mixed by vigorous vortexing with decane. The OD_400 *nm*_ of the aqueous phase was measured and recorded before (A_0_) and after mixing (A_1_). The results showed that absence of *sufCDSUB* resulted in significant increases of surface hydrophobicity with * indicating *P* < 0.05.

Cell surface hydrophobicity is known to significantly influence the ability of the bacterial cells to adhere and colonize a substratum (Krasowska and Sigler, 2014). In an effort to elucidate the factors that underlie the altered biofilm formation in response to the *sufCDSUB* deletion, the cell surface hydrophobicity of the Δ*suf* mutants, along with the wild-type and the complement strain were examined. The results showed that relative to the wild-type, the Δ*suf* mutants, Δ*suf::kan^*r*^* and Δ*suf::erm^*r*^* both had a significantly increased hydrophobicity (*P* < 0.05) (Figure 8). As expected, complementation in Δ*suf* + reduced the hydrophobicity to a level more similar to the wild-type, UA159. This result suggests that cell surface hydrophobicity may play a role in the altered biofilm formation by the Δ*suf* mutants.

### The *sufCDSUB* Cluster Is Co-transcribed

The *sufCDSUB* cluster is predicted to be co-transcribed. To prove if it is actually the case, total RNA was extracted from mid-exponential phase UA159 cultures, and reverse transcription was carried out using a reverse primer complementary to a region about 100 nucleotides into the coding sequence of *sufB*, the last of the cluster (Supplementary Table 1). The resulting cDNA was then used as the template for PCR amplifications with forward primers that anneal around 50 nucleotides upstream of *sufC, -D, -S, -U*, and -*B* gene and with the reverse primers binding to a region 100 nucleotides into the coding region of the respective gene (Supplementary Table 1). The results of the PCR reactions showed that the cDNA allowed amplification of all five genes with the amplicons identical in size to those with genomic DNA as the template control (Supplementary Figures 10A,B). No PCR products were observed when no SuperScript^TM^ III was added in the reverse transcription. These results suggest that the same cDNA contains all five genes, in support of the prediction that the *sufCDSUB* cluster is indeed co-transcribed as an operon, although it is still possible that additional promoters exist within the cluster.

### *SufCDSUB* Expression Is Up-Regulated in Response to Iron Limitation and Oxidative Stress

**FIGURE 9:**
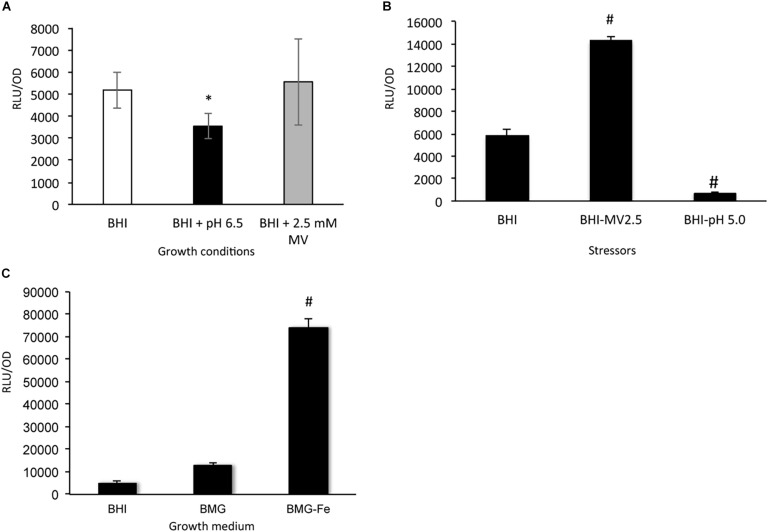
*SufCDSUB* expression by reporter fusion assays. **(A)** Luciferase reporter activity in *Streptococcus mutans* wild-type, UA159 at mid-exponential phase (OD 0.4) was evaluated when grown in BHI with pH adjusted to 6.5 (black) and BHI with the inclusion of methyl viologen (2.5 mM, final conc., gray) simulating acid and oxidative stressors, respectively and compared to regular BHI (white). **(B)** When the reporter strain, UA159 carrying the luciferase reporter, was first grown in regular BHI and then transferred to BHI with methyl viologen at 2.5 mM or BHI adjusted to pH 5.0 and incubated for one hour, methyl viologen was shown to increase reporter activity, while pH 5.0 reduced the reporter activity. #, *P* < 0.01 vs BHI control. **(C)** To test the effects of iron-limitation on *suf* expression, the reporter strain was evaluated in regular BHI (BHI) and BMG (BMG) and BMG without ferric chloride (BMG-Fe). Results showed that transfer from Fe sufficient medium like BMG to Fe-limiting medium BMG-Fe resulted > 5-fold increases (*P* < 0.001). *, *P* < 0.05 vs BHI; #, *P* < 0.001 vs BMG.

Luciferase reporter fusion assays were used to study the regulation of *sufCDSUB* expression with the reporter strain carrying the *sufCDSUB* promoter-reporter grown in regular BHI, BHI with pH adjusted to 6.5, and BHI plus methyl viologen at 2.5 mM, a sub-inhibitory concentration, simulating acid and oxidative stressors, respectively (Figure 9A and Supplementary Figure 11). Compared to the reporter strain grown in regular BHI, significant reductions in luciferase reporter activity were measured at mid- (OD ∼0.4; Figure 9A and Supplementary Figure 11B) and late- (OD ∼0.6; Supplementary Figure 11C) exponential phase (*P* < 0.05), but not during early-exponential phase (OD ∼0.2; Supplementary Figure 11A), when grown in BHI adjusted to pH 6.5. No significant differences in reporter activity were measured when the bacteria were grown in the presence of methyl viologen. However, when the bacterium was first grown in BHI to OD ∼0.2, then transferred to BHI containing MV at 2.5 mM or BHI adjusted to pH 5.0 and allowed to incubate for one hour, > 2-fold increase in luciferase activity was measured with BHI-MV 2.5 mM, while > 7-fold reduction was seen when treated with BHI-pH 5.0 (*P* < 0.01) (Figure 9B).

To analyze the role of iron availability in *sufCDSUB* expression, semi-defined biofilm medium BMG with and without inclusion of ferric chloride (BMG-Fe) (Loo et al., 2000; Wen and Burne, 2002) was used. When the reporter strain was first grown in BMG and then transferred to BMG-Fe, the luciferase reporter activity was elevated by 5.42 (± 1.42)-fold (*P* < 0.001), as compared to that in cells grown in regular BMG medium, which besides ferric chloride also contains casamino acids as a likely source of iron, and in BHI medium (Figure 9C). However, no such differences were observed between the ones grown in BMG and those continuously transferred in BMG-Fe (data not shown). Inclusion of 2,2′-dipyridyl, an iron chelator (at 0.1 mM final concentration, Sigma) in BMG-Fe also led to elevation of the reporter activity by > 3.5-fold, but no effect was observed when the same amount of 2,2′-dipyridyl was added to BMG (data not shown). These results suggest that exposure to iron limitation in the growth environment triggers up-regulation of *sufCDSUB* expression and that *S. mutans* possesses strong capacity to scavenge the trace mineral and adapt to the environment.

## Discussion

The results presented here showed that allelic exchange mutations of the *sufCDSUB* cluster encoding the Fe-S biogenesis system significantly impaired the ability of the deficient mutants to grow under different environmental conditions tested including low pH and in the presence of oxidative and nitrosative stressors. In comparison to the wild-type, the *sufCDSUB*-deficient mutants also demonstrated an increase in mutation frequency and an elevated level of extracellular proteins and DNA. As compared to the wild-type, the ability of the mutants to form biofilms were also reduced significantly. These results suggest that *sufCDSUB* in *S. mutans* plays important roles in regulation of cellular metabolism, cell envelope stability, stress tolerance responses and biofilm formation, traits critical for the pathophysiology of this oral pathogen.

It is apparent that the absence of *sufCDSUB* in *S. mutans* causes major defects in various cellular processes, but the *sufCDSUB* deficient mutant is viable, consistent with the recent observations by Galvao et al. (2015). Multiple systems exist in different bacterial species for the assembly of Fe-S clusters, which play important roles in various cellular processes (Fontecave et al., 2008). *S. mutans* contains only one gene in *sufU* for a homolog of NifU/IscU of the NIF and ISC system, although it possesses multiple genes, including SMU.1051, SMU.841 and *sufS*, which encode proteins with homology to the cysteine desulfurase (Ajdic et al., 2002). From analysis of the flanking regions, these cysteine desulfurase homologs appear to be part of the biosynthetic pathways for other sulfur-containing compounds such as thioRNAs/thiouridine and thiamine. However, it awaits further investigation if in the absence of SufS, one or both of these homologs can utilize cysteine and serve as the source of sulfur. At the meantime, the fact that mutants having the whole *sufCDSUB* cluster deleted remain viable further suggests that the SUF system in *S. mutans* is non-essential, which is not known in any other bacterium lacking the NIF and/or ISC system.

Iron-sulfur-containing proteins IspH and IspG in *B. subtilis* and in *E. coli* are known to play an essential role in the methylerythritol 4-phosphate (MEP) pathway of isoprenoid biosynthesis (Rohdich et al., 2003; Julsing et al., 2007), which is required for production of various bioactive molecules vital in multiple cellular processes. However, recent studies in both *E. coli* and *B. subtilis* have shown that reengineering of the isoprenoid biosynthetic pathway by inserting the mevalonate pathway allows the engineered strains to bypass the two Fe-S containing proteins counteracting the indispensability of the Fe-S cluster biosynthetic systems (Tanaka et al., 2016; Yokoyama et al., 2018). *S. mutans* does not contain IspH and IspG, but does seem to possess a mevalonate pathway (Ajdic et al., 2002). *S. mutans* and several other Gram-positive bacteria, including *S. aureus* incorporated radiolabeled mevalonate into isoprenoids, suggesting that these bacteria use the mevalonate pathway for the synthesis of isoprenoids (Wilding et al., 2000). Therefore, the presence of such a mevalonate pathway suggests a possible alternate pathway(s) in isoprenoid biosynthesis in *S. mutans* and explains in part why the SUF-deficient mutant is viable. However, it is also possible that novel Fe-S assembly systems exist in this bacterium.

Exposure to environmental stressors, especially low pH has been well documented to induce the accumulation of damaged DNA, which affects the growth, metabolism and virulence traits of the bacterial pathogens (Lemos et al., 2005; Lemos and Burne, 2008). In response, the bacterial cells launch stress tolerance responses including elevated expression of enzymes to repair the damaged DNA (Quivey et al., 2001). In *S. aureus*, the DNA repair enzymes MutY (Porello et al., 1998), Nth (Fu et al., 1992) and AddAB (Yeeles et al., 2009) have been shown to require an Fe-S cluster for function. These enzymes are capable of repairing oxidized bases and mismatch mutations throughout the genome, including mutations in *rpoB*, which encodes the beta subunit of RNA polymerase (Roberts et al., 2017). Consistently, the *S. mutans sufCDSUB* mutant was shown to have an increased numbers of rifampicin resistant mutants, which could be in part attributed to the increased mutation frequency in *rpoB* among others. Similarly, when the bacterium is grown in the presence of stressors like low pH and presence of oxygen radical species, where the bacterium is undergoing constant DNA error-repair processes, absence of *sufCDSUB* can directly lead to compromises in DNA repair and consequently, increased mutations and weakened tolerance to acid and oxidative and nitrosative stress responses.

Resistance to Congo Red by *S. aureus* has been attributed to the presence of wall teichoic acids (Suzuki et al., 2012). *S. mutans* is not known to possess cell wall-associated teichoic acids, but it does have rhamnose-containing glucose polymers as the major cell wall associated polymers. Our results showed that the absence of *sufCDSUB* in *S. mutans* led to increased susceptibility to Congo Red (Figure 7A), indicative of likely compromises in cell envelope integrity as a result of *sufCDSUB*-deficiency. In support of this notion, more extracellular DNA and proteins were measured in the cell free culture supernatants of Δ*suf::kan^*r*^* biofilms, compared to the wild-type. Weakened cell envelope is known to lead to increases in the release of eDNA in *S. mutans* and other bacteria (DeFrancesco et al., 2017; Nilsson et al., 2019). Also, further studies are warranted to identify whether the absence of *sufCDSUB* may alter the activity of cell surface proteases involved in processing and conformation of extracellular proteins, which may be involved in the release of these macromolecules.

Iron-sulfur clusters are known to be required for the function of enzymes of many biosynthetic pathways including the synthesis of some branched chained amino acids (Roberts et al., 2017). While the absence of *sufCDSUB* in *S. mutans* is viable, which is unlike *S. aureus, B. subtilis* and others that have been studied, the deficient mutants do display major growth defects especially in media lacking isoleucine, leucine, glutamate and glutamine (Figure 2), whose biosynthesis require Fe-S clusters. Certain Fe-S cluster requiring proteins involved in biosynthetic pathways in *E. coli*, such as LipA, BioB, ThiH, NadA, MoaA, FadH, Fdx and ErpA (see Supplementary Table 3 for more details), were not found in *S. mutans* (Ajdic et al., 2002). These results further suggest the existence of major differences in the SUF system and the Fe-S associated functions among the different bacterial species.

Biofilm formation by *S. mutans* is known to be highly regulated in response to environmental cues (Lemos et al., 2019). The results presented here have shown that the absence of *sufCDSUB* causes major compromises in biofilm formation including alterations in the architecture and reduction in thickness and biovolume. Certainly, the growth defects observed with the *suf* mutants is a major contributing factor. Among other likely factors are the significantly reduced ability of the *sufCDSUB-*deficient mutants to cope with acid and oxidative stressors in the growth environment and the likely compromises in cell envelope integrity, as indicated by the increased extracellular DNA and protein in the biofilm culture medium and the alterations of cell surface hydrophobicity. Cell envelope integrity as well as its physical properties such as distribution and proper conformation of the surface associated proteins can significantly influence bacterial adherence and intercellular interactions, affecting biofilm initiation and biofilm development.

*Streptococcus mutans* is featured with a sucrose-dependent pathway of biofilm formation which involves production of adhesive glucans by GtfB&C with sucrose as a substrate and glucan binding (Bowen and Koo, 2011). Interestingly, more severe compromises appear to be associated with cultures grown in BMGS and less so in BM with rich sucrose (BMS). It suggests that such compromises likely have little to do with the sucrose-dependent biofilm formation. In support of this notion, Western blot analysis also revealed no significant differences in expression of GtfB and GtfC between the Δ*suf::kan^*r*^* mutant and the wild-type when probed using anti-GtfB and anti-GtfC monoclonal antibodies (Data not shown). No significant differences in the EPS production were observed between 24-h biofilms of the mutant and its parent strain when examined using confocal microscopy (Data not shown).

*Streptococcus mutans* is known for its ability to survive low pH and launch an adaptive acid tolerance response following initial exposure to low pH environment, which is featured with enhanced tolerance against lower pH and probably oxidative stressors (Lemos and Burne, 2008). Recent study by Guo et al. have shown that the surface-associated EPS also functions as a proton sinker, and the sinking protons can trigger the adaptive acid tolerance responses, which includes expression of genes for proton translocation and protein repair and results in changes in cell membrane fatty acid composition and likely the surface hydrophobicity (Guo et al., 2015). Likely, when the bacterium is grown in BMS with rich sucrose, more EPS are produced than in BMGS, which confers a higher capacity for retention of protons. Considering the fact that the *suf* mutants can still launch adaptive acid tolerance responses, growth in BMS suggests better capacity of the bacterium to tolerate and survive low pH and likely oxidative stressors, minimizing the defects in biofilm formation, which likely in part explains why more significant compromises in biofilm formation were associated with BMGS and BMG.

The expression of the *sufCDSUB* cluster in several bacteria is highly regulated in response to environmental cues (Outten et al., 2004; Roche et al., 2013). Similarly, environmental stressors such as low pH, oxidative stresses induced by methyl viologen and iron limitation also play a significant role in regulation of *sufCDSUB* expression in *S. mutans*. Like *Synechocystis* sp. PCC 6803 (Vuorijoki et al., 2016) and consistent with its role in Fe-S cluster assembly, *sufCDSUB* expression was elevated significantly when the bacterium was exposed to iron-limiting conditions, such as transfer from regular BMG to BMG-Fe or with addition of proper amount of iron chelator to the culture medium. Interestingly, adaptation to Fe limitation as a result of continuous transferring in BMG-Fe leads to loss of such a response, which is again like *Synechocystis* sp. (Vuorijoki et al., 2016). In *Synechocystis* sp. PCC 6803, the expression of *sufBCDS* is governed by transcriptional repressor SufR that is located in front of *sufBCDS* operon in opposite orientation (Vuorijoki et al., 2016). *S. mutans* possesses no apparent SufR. However, recent study by Galvao *et al.* showed that *sufCDSUB* is part of the Spx regulon (Galvao et al., 2015). During the preparation of this manuscript, Kajfasz *et al.* have also provided evidence that PerR, a member of the Fur (for ferric uptake repressor) family of regulators, plays a direct role in regulation of *sufCDSUB* expression (Kajfasz et al., 2020). Analysis of the *sufCDSUB* promoter region using Virtual Footprint^1^ also identified two regions, AGAAAAAG (nt -78 to -85 relative to the translation initiation site ATG) and TGATAAAT (nt -16 to -23) with high similarity to the Fur-box, the binding site for Fur in *E. coli* (Troxell and Hassan, 2013). Study is under way to uncover if and how the different elements govern the expression of *sufCDSUB* under different environmental conditions.

Iron homeostasis and its regulation have been shown to play an important role in various cellular processes including biofilm formation. In *E. coli*, biofilm formation is reduced during iron starvation or in the absence of the ISC cluster assembly system. Deletion of regulator IscR leads to increased biofilm formation through induction of type I fimbriae encoded by the *fimAICDFGH* operon (Wu and Outten, 2009). In *P. aeruginosa*, iron limitation can reduce biofilm formation by blocking early steps in microcolony formation (Banin et al., 2005). In *S. mutans*, our previous studies have shown that Fe limitation influences bacterial growth and causes alterations in cell morphology (Liao et al., 2014). As an Fe-S machinery, *sufCDSUB-*deficiency will affect the Fe-S dependent circuits, which is known to play important roles in various biological functions such as substrate binding/activation, transcriptional and translational regulation, and as cofactors in essential biochemical pathways (Johnson et al., 2005), consequently influencing growth, stress tolerance responses, and biofilm formation as were displayed by the *suf* mutants.

In an initial characterization of the *sufCDSUB* cluster, Galvao et al. (2015) demonstrated several observations that are highly consistent to what were observed in this study including sensitivity to oxidative stress and a reduced growth rate of a *sufCDSUB* deficient mutant compared to the wild-type. Interestingly, the study also showed that unlike the result presented here, the *suf* mutant had higher level of biofilms compared to the wild type, although that was done with *S. mutans* strains grown in BM medium supplemented with 1% sucrose (Galvao et al., 2015). Under similar conditions, we observed no significant differences in the biofilm formation between the wild-type and the *suf* mutant (data not shown). Likely, these differences can also be in part attributed to how the *suf* mutants were constructed. The *suf* mutant in the Galvao et al. study was constructed by using a polar Ω kanamycin resistance cassette to replace portion of the *sufC-*coding sequence. The Ωkan cassette is known to have a transcriptional terminator in its 3′ end, which when inserted is presumably able to block transcription of the downstream genes under the direction of the upstream promoter. As shown by our reverse transcription-PCR, the *sufCDSUB* cluster is transcribed as an operon, as predicted (Ajdic et al., 2002). However, the possibility that some of the downstream genes can still be transcribed separately under certain conditions cannot be excluded. In support of this notion, a more recent RNA-seq study by Kajfasz et al. (2017) also showed that the transcript level of *sufB* was 30% less than some other members of the cluster following exposure to 0.5 mM hydrogen peroxide for 5 min (Kajfasz et al., 2017), although if and how the *sufCDSUB* cluster is differentially expressed await further study. It is also worthy noting that while the SufCDSUB system likely functions as a whole complex in this bacterium, it is also well documented that SufB and SufC are the minimal core of the Fe-S cluster biogenesis system. As indicated above, there is a possibility that certain components like SufS can be compensated in the event of its deficiency. These could be part of the contributing factors to the observations that the mutants with individual component deleted/inactivated do not phenocopy to one another and those with deletion of the whole system. However, the exact underlying mechanisms await further investigation.

It is apparent that differences in the structure and function exist between the SUF systems in *S. mutans* and other major bacterial species that have been studied. Similar to the *E. coli* SUF system, the *S. mutans* SUF system plays an important role in oxidative stress tolerance responses and the *sufCDSUB* expression is up-regulated in response to oxidative stressors and Fe availability in the environment. However, like *S. aureus* and *B. subtilis* but unlike *E. coli, S. mutans* does not have the *sufA* gene, which in *E. coli* encodes a scaffold protein for Fe-S cluster synthesis and also described as an Fe-S cluster carrier (Ollagnier-de-Choudens et al., 2004; Chahal and Outten, 2012). On the other hand, the SUF system in *S. aureus* was shown to be essential, where the knock-down mutants were unable to survive under the conditions studied (Roberts et al., 2017), unlike what is observed with *S. mutans* strains lacking the *sufCDSUB*. It was also shown that a *S. aureus* strain defective in Fe-S protein maturation resulted in increased biofilm formation and decreased production of exoproteins (Mashruwala et al., 2016), observations that are different from *S. mutans.* However, the absence of *sufCDSUB* in *S. mutans* also leads to phenotypes a lot similar to what were observed in *S. aureus*, including increased sensitivity to oxidative and nitrosative stressors and growth defects especially in the absence of specific amino acids (Roberts et al., 2017). Like *S. mutans*, the *S. aureus* SUF system also has five major components, in SufC, -D, -S, -U, and -B (Roberts et al., 2017). In *S. aureus*, SufU acts as a sulfur transfer protein that provides the sulfur to SufBCD, which is a molecular scaffold for Fe-S cluster synthesis (Wollers et al., 2010). Phylogenetic analysis and multiple sequence alignment using EBI-EMBL Clustal Omega^2^ using the primary amino acid sequences of the SufU proteins further showed that *S. mutans* SUF shares high similarity with other streptococci and members in the firmicutes phylum with the highest similarity measured with *S. sobrinus* and *S. agalactiae*, and less identity was observed with *P. aeruginosa* and *E. coli* (Supplementary Figures 12, 13). These results further suggest that similarity and differences in SUF structure and function exist among different species within the firmicutes phylum.

In summary, the present study has demonstrated that *sufCDSUB* in *S. mutans* plays important roles in various cellular processes including glycolysis and amino acid biosynthesis, growth, environmental stress tolerance responses and biofilm formation, although the mutants deficient of *sufCDSUB* are viable. These results further suggest that the SUF system of Fe-S cluster assembly in *S. mutans* is non-essential, although it awaits further investigation how the *sufCDSUB* mutants compensate the deficiency of Fe-S clusters in regulation of the bacterial physiology and if any additional novel Fe-S assembly systems exist in this bacterium.

## Data Availability Statement

The raw data supporting the conclusions of this article will be made available by the authors, without undue reservation.

## Author Contributions

ZW and JB conceived the experiments. KE, XH, RR, and ZW conducted the experiments and analyzed the data. KE, JB, and ZW wrote the manuscript. All authors contributed to the article and approved the submitted version.

## Conflict of Interest

The authors declare that the research was conducted in the absence of any commercial or financial relationships that could be construed as a potential conflict of interest.

## Footnotes

1 http://www.prodoric.de/vfp/

2 www.ebi.ac.uk/Tools/msa/clustalo/
