# Supplementary material for: Streptococcus mutans Lacking sufCDSUB Is Viable, but Displays Major Defects in Growth, Stress Tolerance Responses and Biofilm Formation
Source: Front Microbiol. 2021 Jun 24;12:671533. doi: 10.3389/fmicb.2021.671533 (PMC8264796; doi:10.3389/fmicb.2021.671533)
Supplement: Supplementary file 1 [file Data_Sheet_1.pdf]

Streptococcus mutans Lacking _sufCDSUB_ Is Viable, But Displays Major Defects in Growth, Stress Tolerance Responses and Biofilm Formation

(supplemental data)

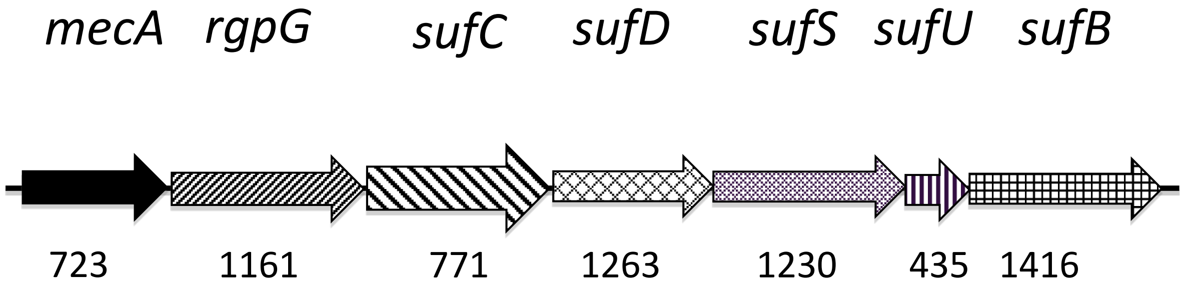

Figure S1. The schematic diagram of the _suf_ cluster and flanking regions. The *S. mutans* SUF Fe-S cluster comprises of *sufC*, *-D, -S*, *-U* and *-B,* located immediately downstream of the *uppP-mecA-rgpG* cluster.

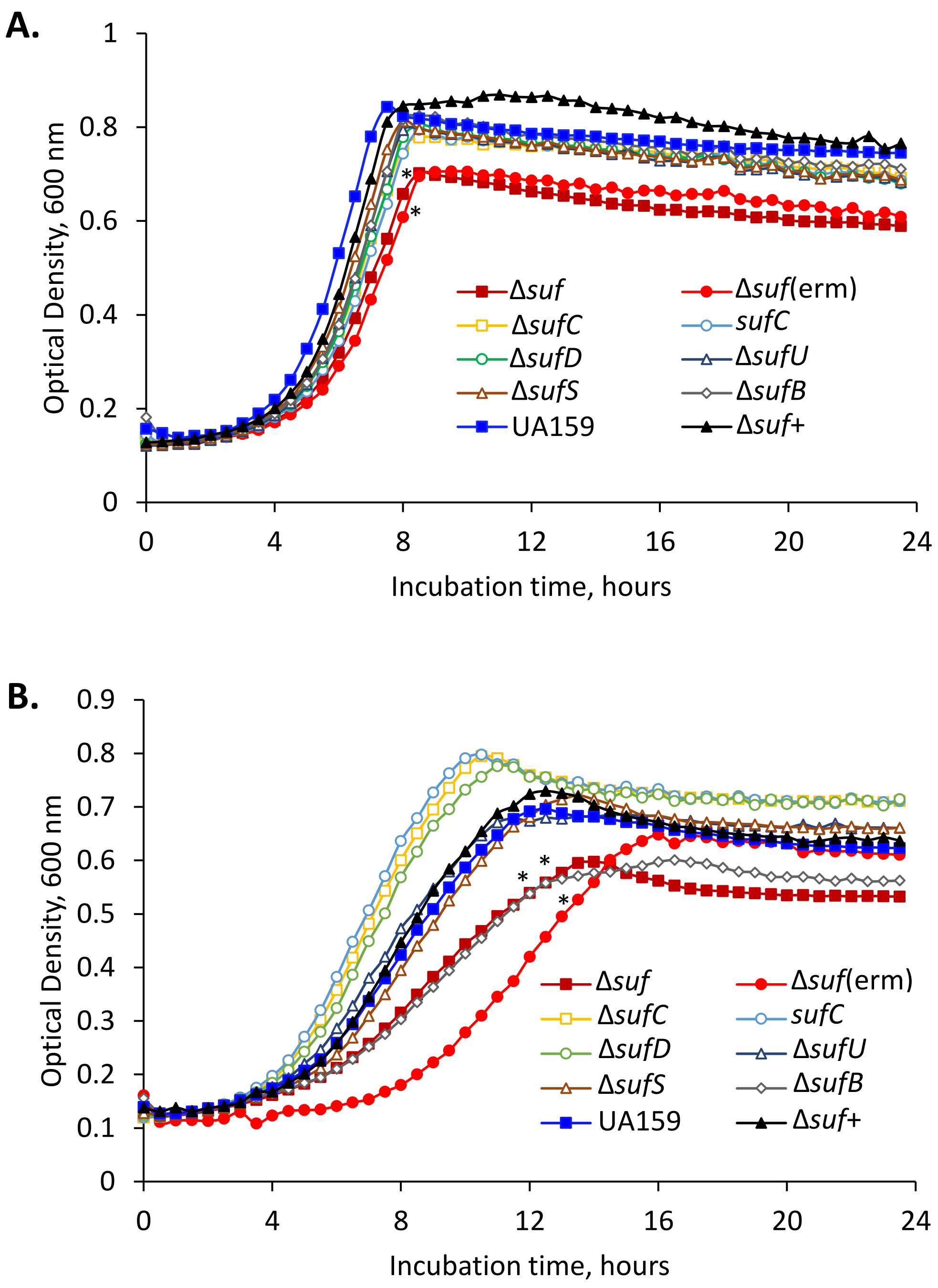

Figure S2. Growth studies. The growth of *S. mutans* wild-type, UA159 and the allelic exchange mutants with deficiency of *sufCDSUB*, individually or as a whole when grown in regular BHI medium (A) and BHI adjusted to pH 6.0 (B) was evaluated continuously using Bioscreen C. In regular BHI, *sufCDSUB* mutants, Δ*suf*::*kan*^{r} (Δ*suf*) and Δ*suf*::*erm*^{r} (Δ*suf*(erm)), had a significantly slower growth rate compared to the wild-type, UA159 (*, *P* < 0.05). Mutants Δ*sufC::spc*^{r} (Δ*sufC*), Δ*sufD::kan*^{r} (Δ*sufD*), Δ*sufS::kan*^{r} (Δ*sufS*), Δ*sufU::erm*^{r} (Δ*sufU*), Δ*sufB::kan*^{r} (Δ*sufB*), and *sufC::kan*^{r} (*sufC*) all demonstrated no significant differences in growth rate, when compared to the wild-type. At pH 6, Δ*suf*, Δ*suf*(erm) and Δ*sufB* mutants displayed a major reduction in growth rate as compared to the wild-type (*, *P* < 0.05), while the growth rates of Δ*sufD*, Δ*sufC*, and *sufC* were increased slightly.

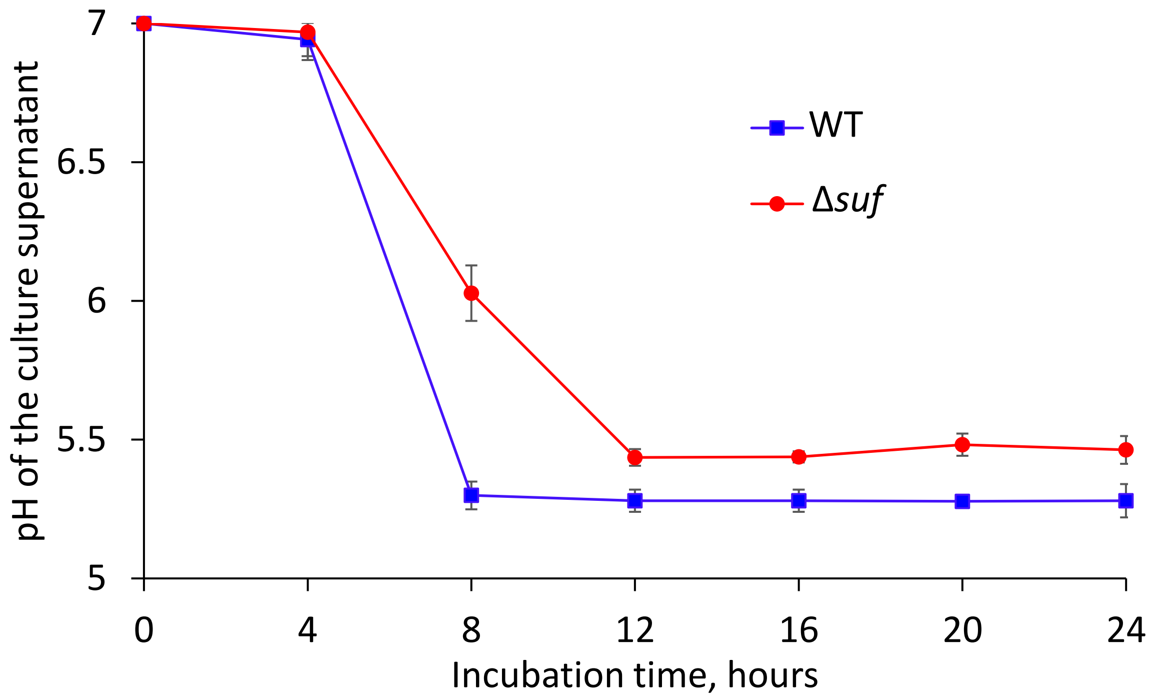

Figure S3. The pH measurements of the supernatants. The culture supernatant pH of *S. mutans* wild-type, UA159 and Δ*suf::kan*[r] mutant, Δ*suf* was measured when grown in regular BHI medium. The results showed that the pH of the Δ*suf* mutants' supernatant did not reduce as much compared to the wild-type.

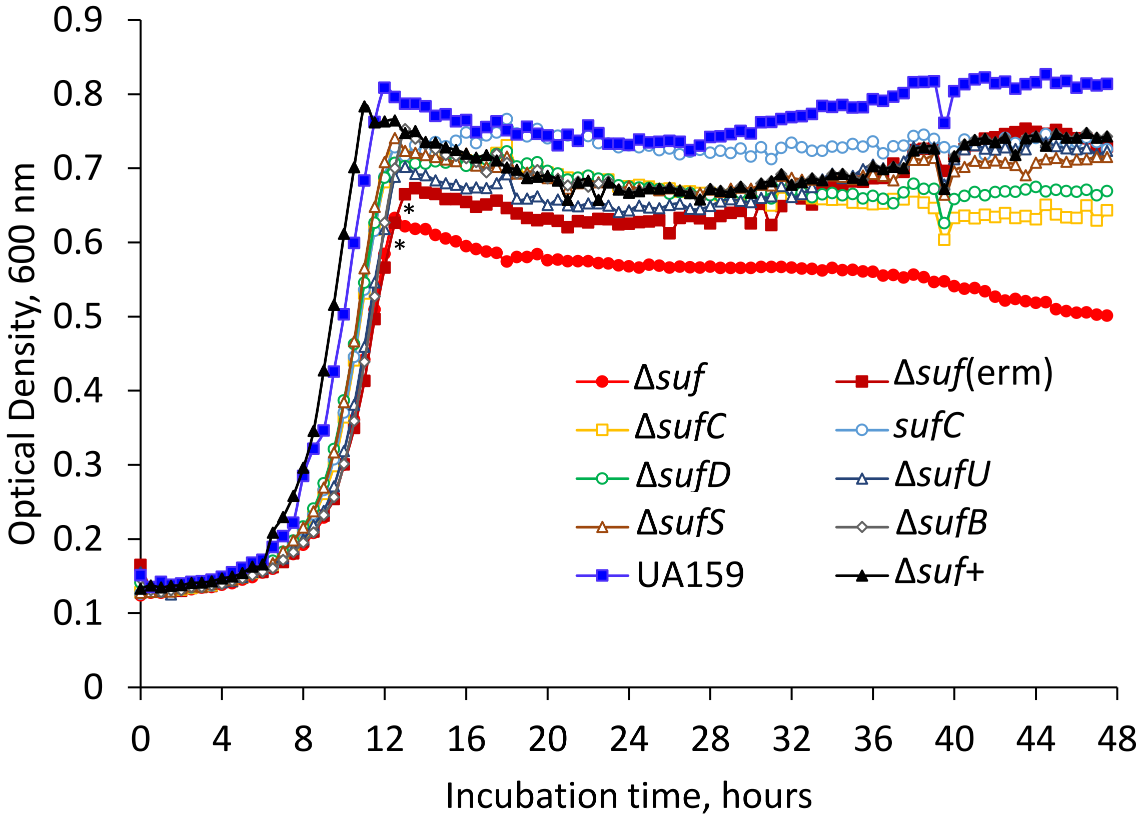

Figure S4. Growth studies in the presence of methyl viologen. The wild-type, *S. mutans* UA159 and the *suf* deficient mutants were grown in BHI broth with addition of 12.5 mM methyl viologen (MV). The data show significant reduction in the growth rate of the *suf* mutants, Δ*suf::kan*^{r} (Δ*suf*) and Δ*suf::erm*^{r} (Δ*suf*(erm)) when compared to the wild-type, UA159 (*, *P* < 0.05). Mutants Δ*sufC::spc*^{r} (Δ*sufC*), *sufC::kan*^{r} (*sufC*), Δ*sufD::kan*^{r} (Δ*sufD*), Δ*sufS::kan*^{r} (Δ*sufS*), Δ*sufU::erm*^{r} (Δ*sufU*) and Δ*sufB::kan*^{r} (Δ*sufB*) also demonstrated slower growth rates compared to the wild-type, but such differences were not statistically significant.

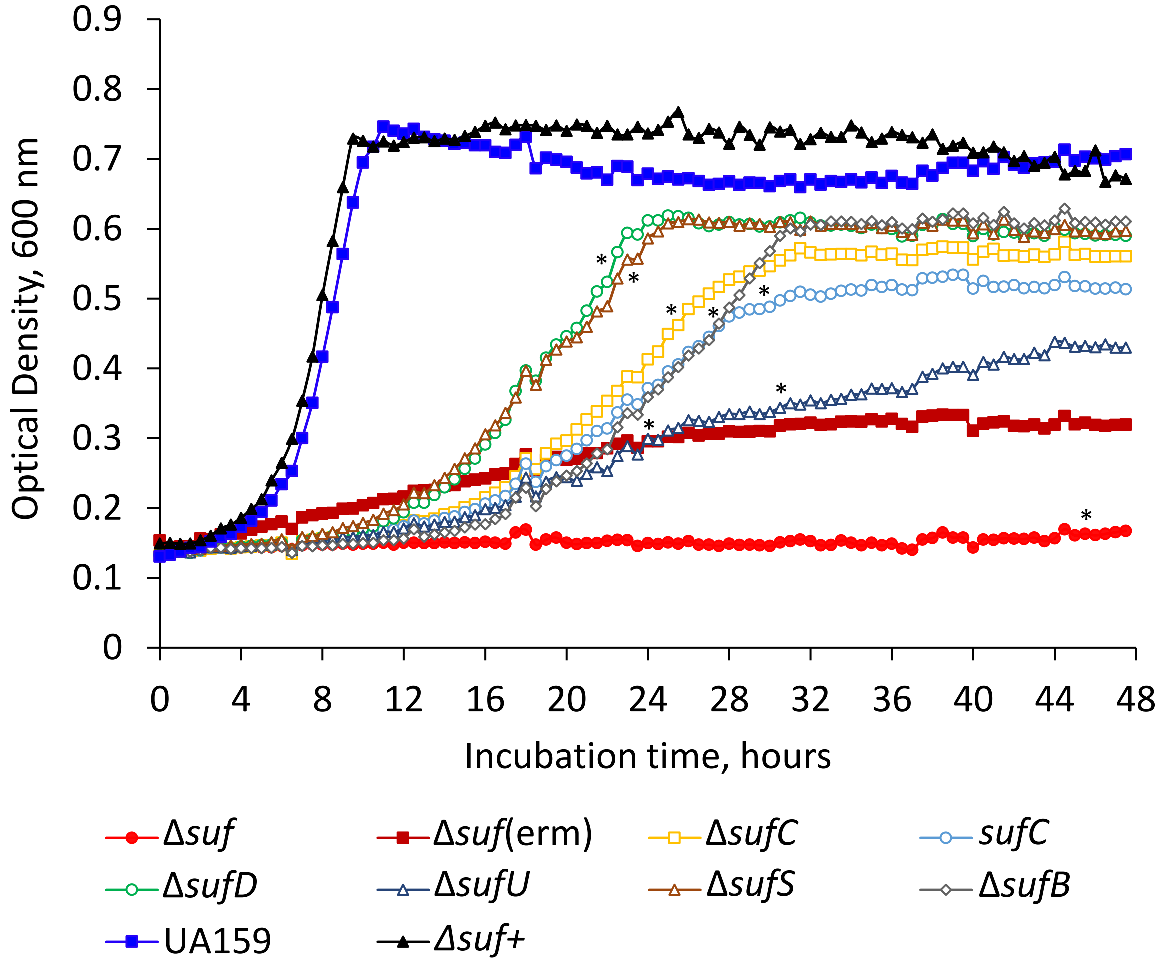

Figure S5. Growth in the presence of sodium nitroprusside. *S. mutans* wild-type UA159 and its *suf* deficient mutants, Δ*sufC::spc*^{r} (Δ*sufC*), Δ*sufD::kan*^{r} (Δ*sufD*), Δ*sufS::kan*^{r} (Δ*sufS*), Δ*sufU::erm*^{r} (Δ*sufU*), Δ*sufB::kan*^{r} (Δ*sufB*), and *sufC::kan*^{r} (*sufC*), and the complement strain, Δ*suf*+ were grown in BHI with inclusion of sodium nitroprusside at 2 mM, final conc., and the results showed that all mutants had a significant reduction in growth rate compared to the wild-type (*, $P < 0.001$).

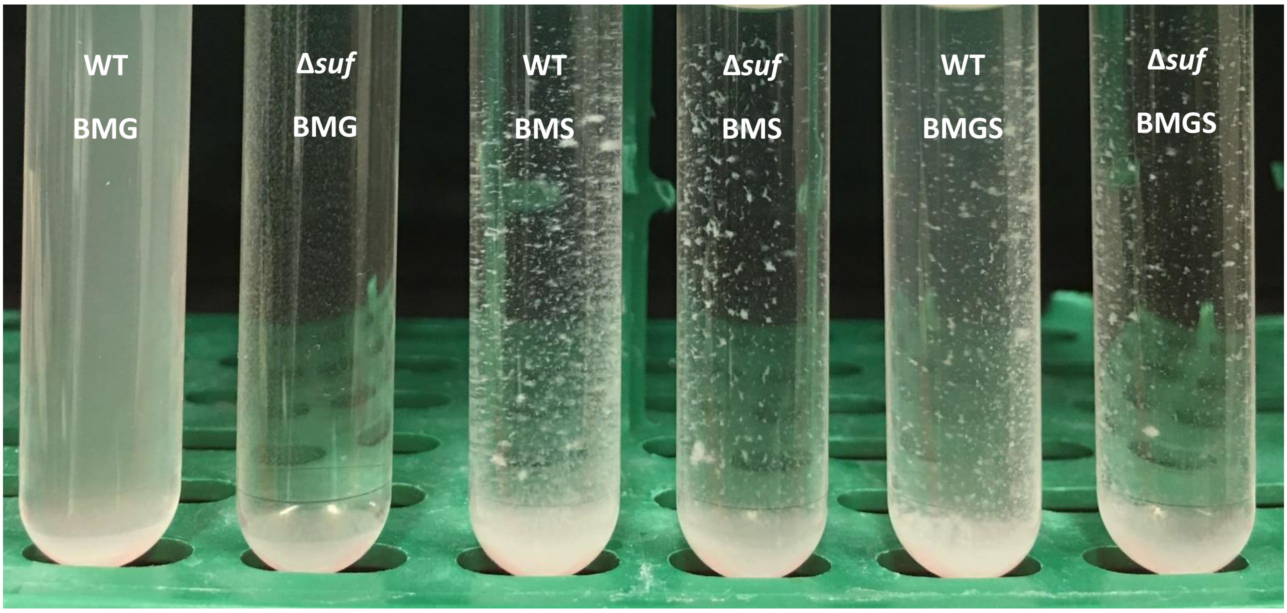

Figure S6. Growth characteristics of the Δ*suf* mutant in biofilm medium. *S. mutans* UA159 and the Δ*suf::kan*^{r} mutant, Δ*suf* were grown in semi-defined biofilm medium (BM) supplemented with glucose (20 mM, BMG), sucrose (20 mM), and glucose plus sucrose (18 mM and 2 mM, respectively, BMGS). In BMG, Δ*suf* mutant formed aggregates in the bottom of the test tube and showed a major reduction in the culture density compared to the wild-type. In BMS, Δ*suf* mutant showed slightly less growth compared to the wild-type. In BMGS, the wild-type had more growth compared to the deficient mutant with both tubes containing large aggregates.

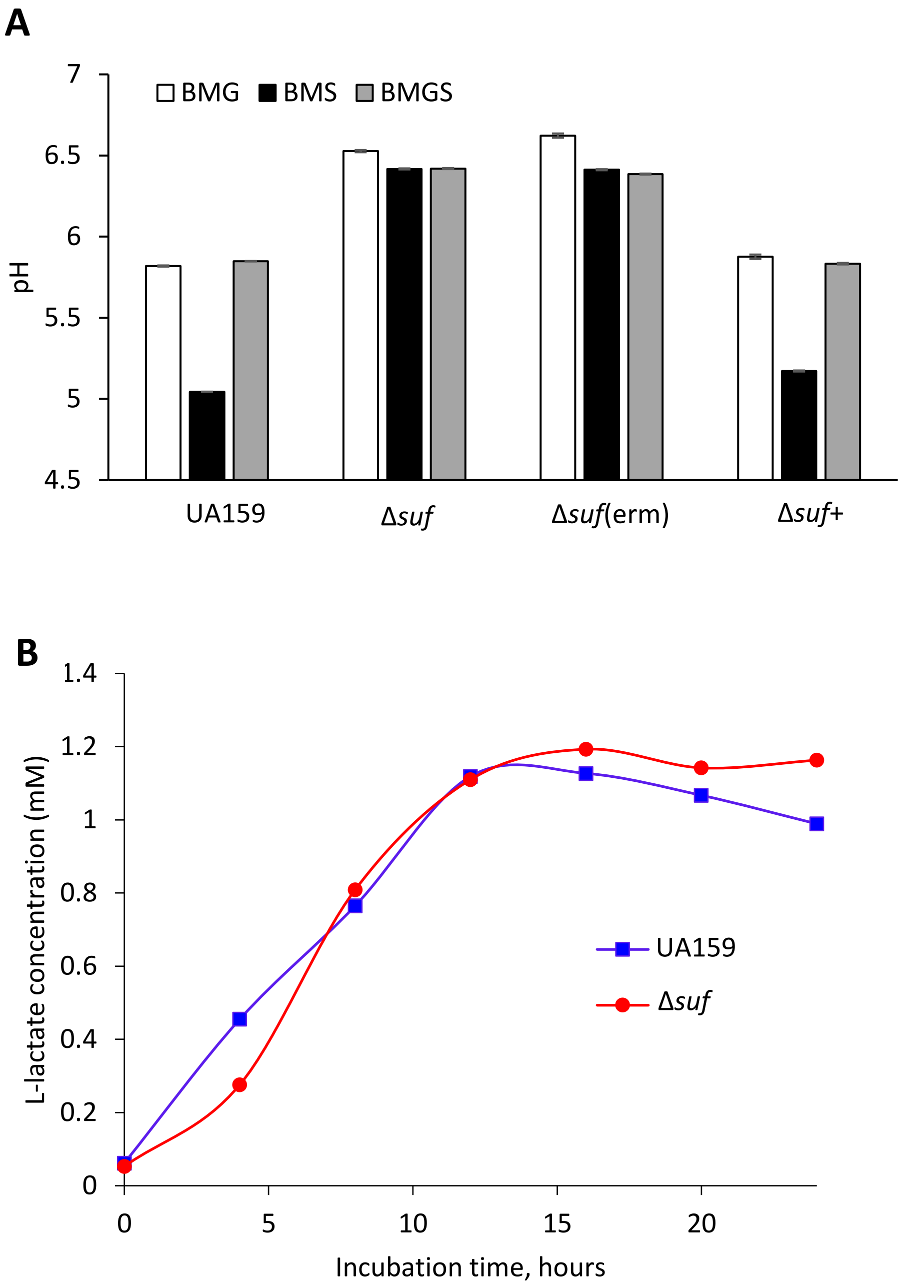

Figure S7. pH and lactic acid profile. (A) The end-point pH of the biofilm supernatants of *S. mutans* wild-type UA159, its *sufCDSUB*-deficient mutants, Δ*suf::kan*^{r} (Δ*suf*) and Δ*suf::erm*^{r} (Δ*suf*(erm)), and the complement strain, Δ*suf*+ was measured after growth for 24 hours in biofilm medium BMG, BMS and BMGS. Mutants Δ*suf* and Δ*suf*(erm) both had a higher culture pH than the wild-type with the highest differences observed when grown in BMS medium. (B) Lactic acid concentration of the cell-free biofilm culture medium of the wild-type, UA159 and Δ*suf* mutant was measured over a period of 24 hours when grown in BMGS medium. The results showed no significant differences in the lactic acid concentration between UA159 and Δ*suf* mutant.

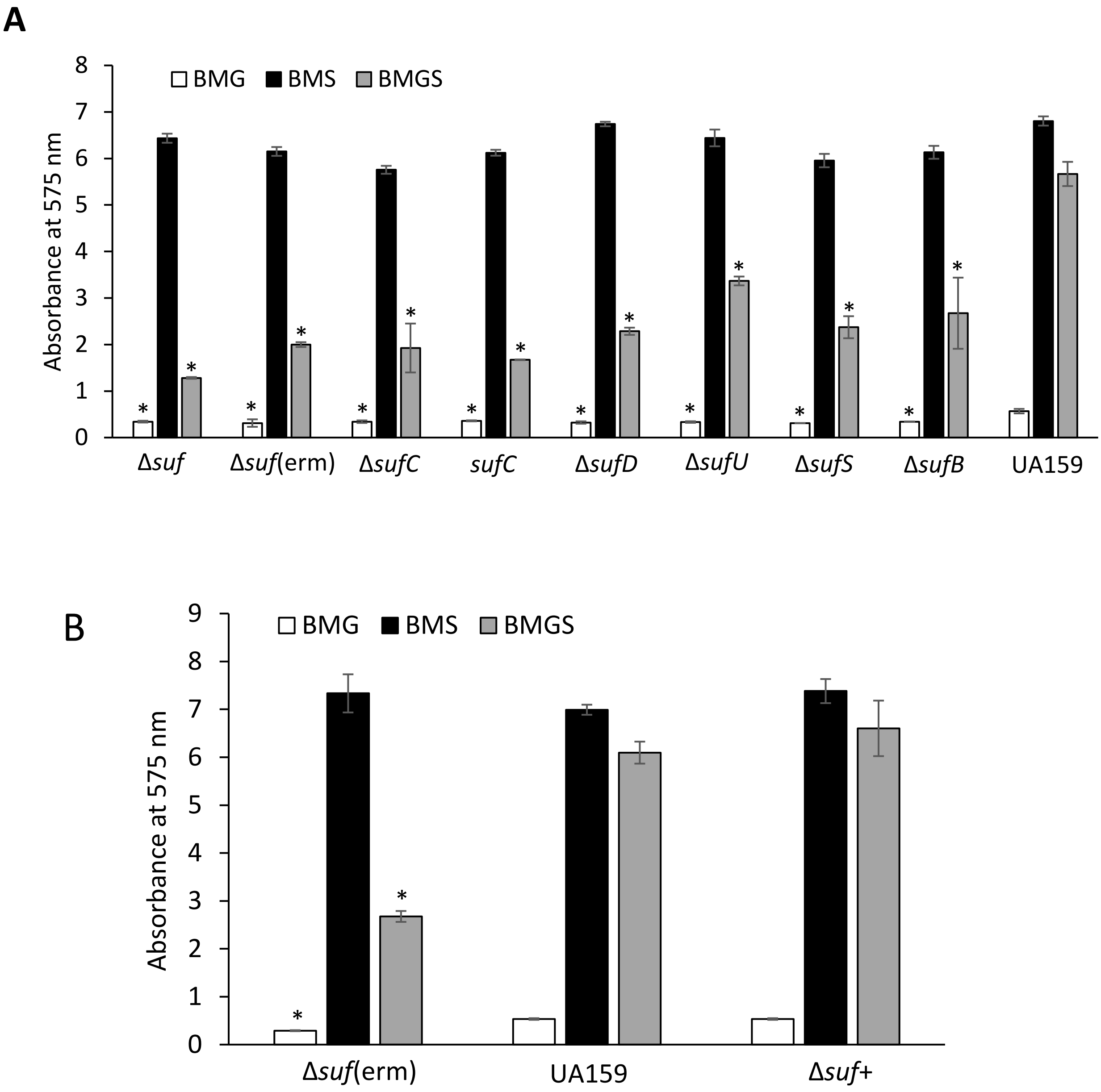

Figure S8. Biofilm formation in 96-well plates. *S. mutans* wildtype UA159, its mutants with deletion /inactivation of *sufCDSUB* individually or as a whole, and the complement strain were grown in 96-well plates in semi-defined biofilm medium (BM) with glucose (20 mM, BMG), sucrose (20 mM), and glucose plus sucrose (18 mM and 2 mM, respectively, BMGS). (A) Relative to the wild-type, the *suf* mutants all formed significantly less biofilms when grown in BMG and BMGS (*, $P < 0.05$), although no significant differences were measured between UA159 and the *suf* mutants when grown in BMS. (B) as expected, the complement strain, Δ*suf*+ restored the biofilm formation to a level similar to the wild-type (*, $P < 0.05$), when grown in the conditions as indicated.

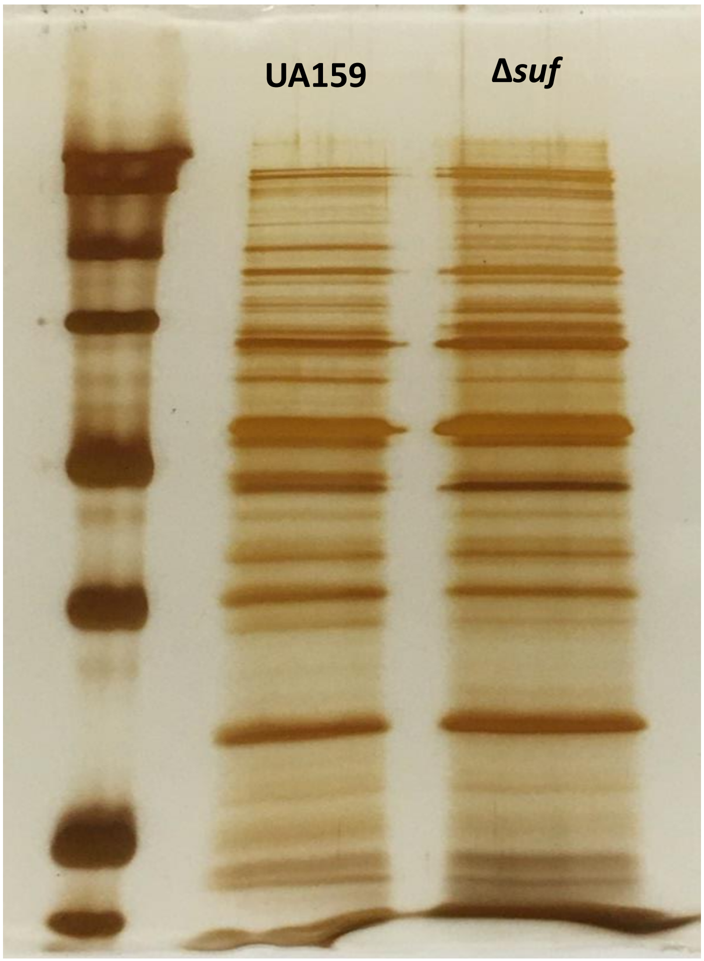

Figure S9. SDS-PAGE analysis of extracellular proteins. Extracellular proteins *of S. mutans* wild-type, UA159 and Δ*suf::kan*[r] mutant, Δ*suf* were purified from 24-hour biofilms, separated using a 12% SDS-PAGE, and stained with a silver staining kit (GE Healthcare). Results showed that the Δ*suf* mutant had significantly more exoprotein content than the parent strain. It required 11-fold more volume of exoprotein preps of the wild-type to achieve a similar level of exoproteins, as shown in the SDS-PAGE.

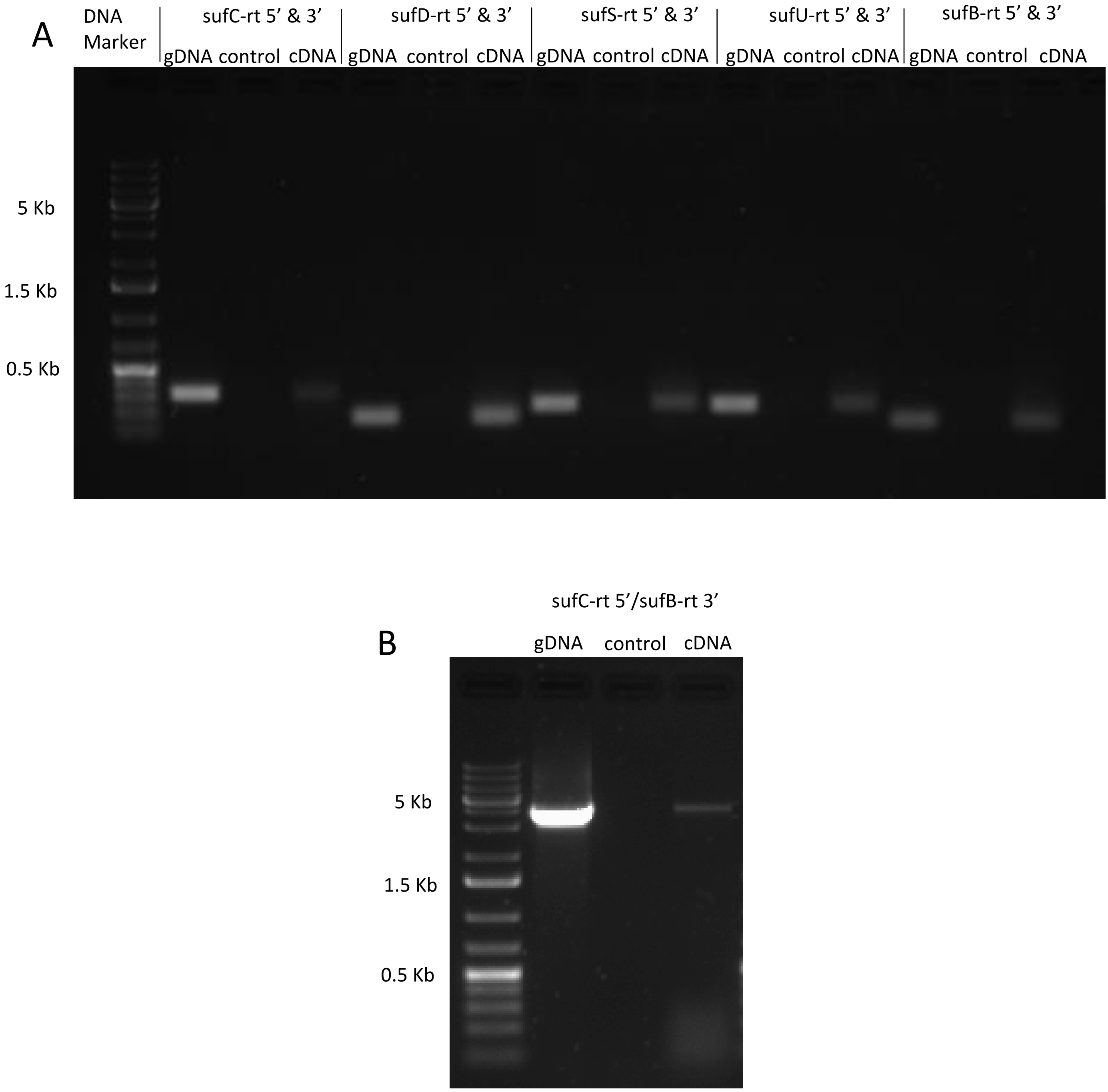

Figure S10. RT-PCR analysis of the *sufCDSUB* cluster. (A) The cDNA synthesized with the sufB-rt reverse primer was PCR amplified with forward and reverse primer pairs that target the respective *sufC, -D, -S, -U,* and *-B*. (B) The synthesized cDNA was PCR amplified using sufC-rt 5' forward primer and the sufB-rt 3' reverse primer. Genomic DNA (gDNA) was used as the positive control, and a RT-PCR reaction without RNA polymerase was used as the negative control. The results showed that the cDNA allowed amplification of all five genes with the amplicons identical to those with genomic DNA as the template control, which further suggests that the cDNA contains all five genes and the *sufCDSUB* cluster is indeed co-transcribed as an operon under the conditions studied.

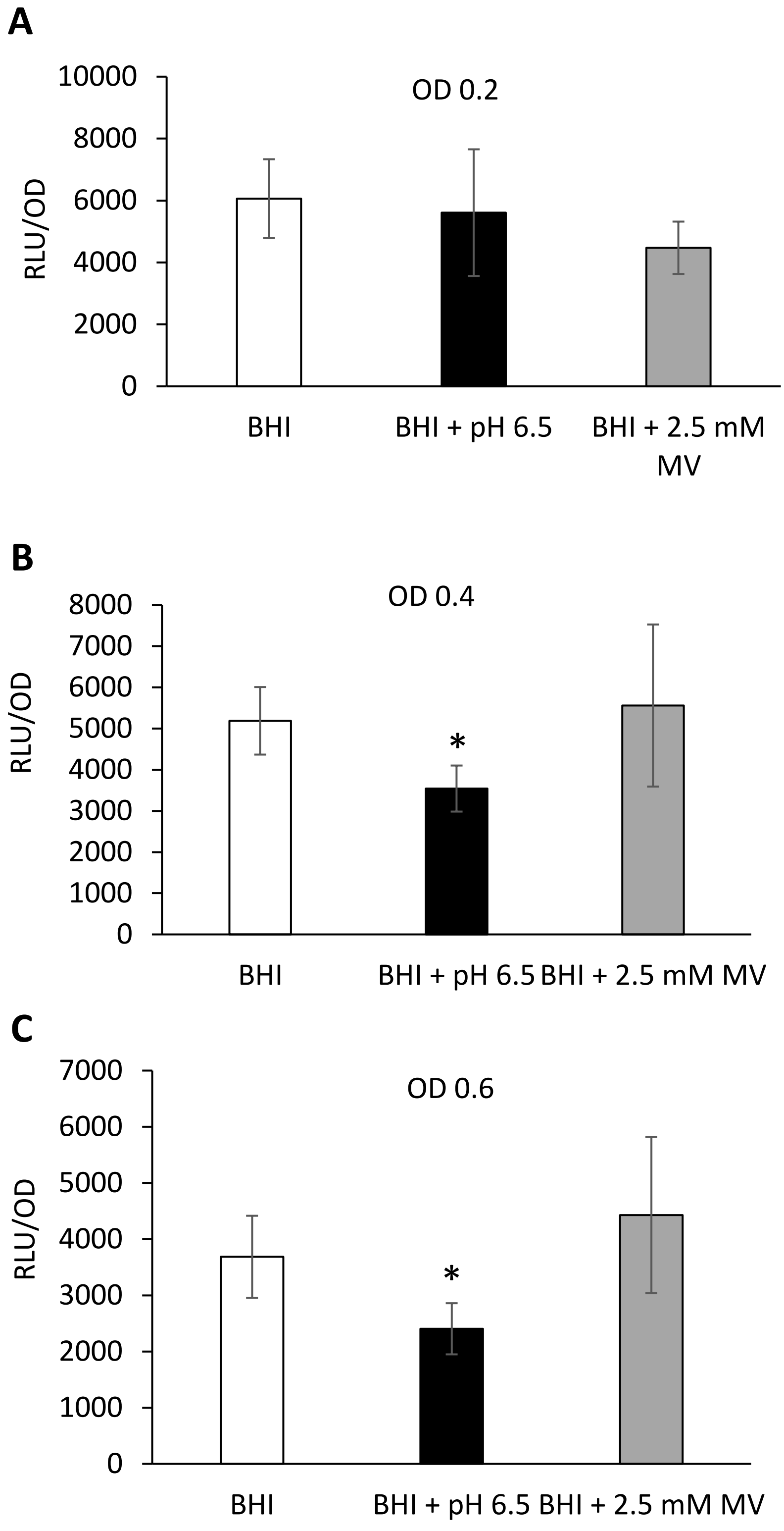

Figure S11. Luciferase reporter analysis. The luciferase reporter activity under the direction of the cognate *suf* promoter was evaluated with the reporter strain grown in regular BHI (white), BHI adjusted to pH 6.5 (black) and BHI with the presence of 2.5 mM methyl viologen (MV) (grey) during early- (OD_{600nm} 0.2; A), mid- (OD_{600nm} 0.4; B) and late- (OD_{600nm} 0.6; C) exponential phase of growth. As compared to regular BHI, the luciferase activity in BHI-pH 6.5 was significantly lower at mid- and late-exponential phase ($P < 0.05$), although no significant differences were measured during early-exponential phase and in the presence of MV at the concentration tested.

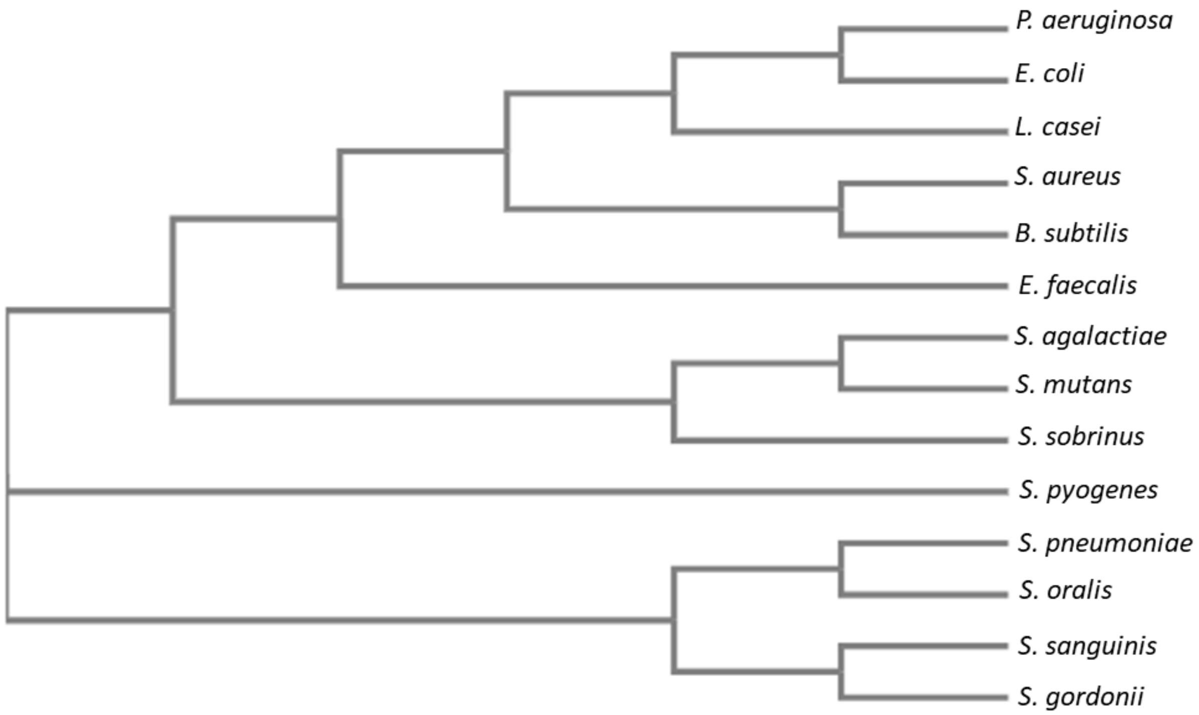

Figure S12. Phylogenetic analysis of SufU proteins. To analyze the similarity of *S. mutans* SUF system with the ones of the other major bacterial species, the SufU primary amino acid sequences of *Pseudomonas aeruginosa, Escherichia coli, Staphylococcus aureus, Bacillus subtilis, Streptococcus pyogenes, Lactobacillus casei, Enterococcus faecalis, Streptococcus pneumoniae, Streptococcus agalactiae, Streptococcus gordonii, Streptococcus sanguinis, Streptococcus oralis, Streptococcus sobrinus* and *Streptococcus mutans* were aligned and further analyzed using EBI-EMBL Clustal Omega (https://www.ebi.ac.uk/Tools/msa/clustalo/) (open source). The *S. mutans* SufU showed the highest similarity to *S. agalactiae*, but interestingly distant from the other streptococci including other members of the oral streptococci analyzed and members in the phylum Gammaproteobacteria including *E. coli* and *P. aeruginosa*. These results also suggests that similarities and differences in SUF structure and likely, function among the different bacterial species analyzed.

	P. aeruginosa	*E. coli*	*L. casei*	*S. aureus*	*B. subtilis*	*E. faecalis*	*S. agalactiae*	*S. mutans*	*S. sobrinus*	*S. pyogenes*	*S. pneumoniae*	*S. oralis*	*S. sanguinis*	*S. gordonii*
P. aeruginosa		49.63	46.08	47.65	47.15	49.01	45.41	46.4	46.02	46.77	46.27	46.52	47.89	46.9
E. coli	49.63		44.16	50.74	46.02	48.51	49.25	48.76	46.77	47.01	49.5	50.25	48.76	48.01
L. casei	46.08	44.16		46.23	48.99	53.02	47.47	47.22	49.49	49.75	46.46	46.97	47.98	48.23
S. aureus	47.65	50.74	46.23		60.84	59.17	56.23	54.66	54.05	53.56	56.76	56.02	56.48	55.75
B. subtilis	47.15	46.02	48.99	60.84		62.81	59.41	58.91	59.41	61.39	61.39	61.63	61.39	61.39
E. faecalis	49.01	48.51	53.02	59.17	62.81		66.67	67.16	70.1	67.89	68.87	69.12	69.36	68.38
S. agalactiae	45.41	49.25	47.47	56.23	59.41	66.67		78	76.72	71.57	72.06	71.81	73.9	72.68
S. mutans	46.4	48.76	47.22	54.66	58.91	67.16	78		78.68	73.77	74.51	75.25	74.82	72.86
S. sobrinus	46.02	46.77	49.49	54.05	59.41	70.1	76.72	78.68		74.26	75	75.74	73.28	72.3
S. pyogenes	46.77	47.01	49.75	53.56	61.39	67.89	71.57	73.77	74.26		75	75.98	75.49	74.26
S. pneumoniae	46.27	49.5	46.46	56.76	61.39	68.87	72.06	74.51	75	75		95.59	83.82	82.84
S. oralis	46.52	50.25	46.97	56.02	61.63	69.12	71.81	75.25	75.74	75.98	95.59		83.82	83.09
S. sanguinis	47.89	48.76	47.98	56.48	61.39	69.36	73.9	74.82	73.28	75.49	83.82	83.82		95.37
S. gordonii	46.9	48.01	48.23	55.75	61.39	68.38	72.68	72.86	72.3	74.26	82.84	83.09	95.37	

Figure S13. Identity analysis of SufU. To examine the structure and function similarity and differences between *S. mutans* SUF and the other major species, the primary amino acid sequences of *S. mutans* SufU were analyzed with the SufU of *P. aeruginosa, E. coli, S. aureus, B. subtilis, S. pyogenes, L. casei, E. faecalis, S. pneumoniae, S. agalactiae, S. gordonii, S. sanguinis, S. oralis,* and *S. sobrinus* using the EBI-EMBL Clustal Omega program (https://www.ebi.ac.uk/Tools/msa/clustalo/) (open source), and the results were presented as percentage of identity when SufU between the different species were compared. As expected, the high similarity exists between *S. mutans* and the other members of the streptococci with the highest measured with *S. sobrinus* and *S. agalactiae,* and the less identity was observed with *P. aeruginosa* and *E. coli*, members of the phylum Gammaproteobacteria. These results again suggest differences and similarities in structure and likely, function exist in the SUF system among different species within the firmicutes phylum.
