# Supplementary material for: Streptococcus mutans Lacking sufCDSUB Is Viable, but Displays Major Defects in Growth, Stress Tolerance Responses and Biofilm Formation
Source: Front Microbiol. 2021 Jun 24;12:671533. doi: 10.3389/fmicb.2021.671533 (PMC8264796; doi:10.3389/fmicb.2021.671533)
Supplement: Supplementary file 2 [file Table_1.pdf]

Table S1. Primers used in this study

Names	Forward (5' to 3')	Reverse (5' to 3')	Application
Suf 5R1	ttcgatgattagtctgaccactatgg	gatgaattctgcattagtaattcctg	5' fragment for Δ*suf::kan*^{r}
Suf 3R1	ctcatacgaattcttctattgtctcc	taccaactaactcttgataactatc	3' fragment Δ*suf::kan*^{r}
Suf 5Xb	ttcgatgattagtctgaccactatgg	gatgaactcagaattagtaattcctg	5' fragment for Δ*suf::erm*^{r}
Suf 3Bm	taaggatcctcataccagttcttctattg	taccaactaactcttgataactatc	3' fragment for Δ*suf::erm*^{r}
SufRp	atgctagcatgctccactttaatcctttc	aactcgagcgctcctttcatttatcattattc	P*suf* reporter fusion
SufC5	tcccatgctccactttaatcctttcttg	gatctagatcatcaacttccatttccaa	5' fragment for Δ*sufC::kan*^{r}
SufC3	aactcgagcacaccagatattgttcatg	tcactcgagtgtcaattccttgaa	3' fragment for Δ*sufC::kan*^{r}
Suf-C	ttcgatgctagctctgaccactatgg	taggtaccaactaactcttgataactatc	*suf* complementation, Δ*suf*+
SufB5	ttggaacagtcattactgaaattcctg	acgaattcatgaaagccaaattgatagtc	5' fragment for Δ*sufB::kan*^{r}
SufB3	ctcatacgaattcttctattgtctcc	taccaactaactcttgataactatc	3' fragment for Δ*sufB::kan*^{r}
SufU5	ttggaacagtcattactgaaattcctg	tattagaattcacctgttccacatcttc	5' fragment for Δ*sufU::kan*^{r}
SufU3	ccttaagaattctattgagcgacaag	atagatttggagacaatagaagaac	3' fragment for Δ*sufU::kan*^{r}
SufD5	ttcgatgattagtctgaccactatgg	taagcgtctagattgtagccaagcag	5' fragment for Δ*sufD::kan*^{r}
SufD3	ttggatccgtcattactgaaattcctgt	tattaagattaacctgttccacatcttc	3' fragment for Δ*sufD::kan*^{r}
SufS5	tatcatacagcttattttaacagtg	tgtctatagaattcattcaaacaag	5' fragment for Δ*sufS::kan*^{r}
SufS3	atgaattcttatttaggtcttcattcag	aacaataccaagtttatcatattcttc	3' fragment for Δ*sufS::kan*^{r}
SufU-rt	atcaattatttaggtcttcattcag	tattaagattaacctgttccacatcttc	RT-PCR of *sufU*
SufB-rt	ttaagaaagctattgagcgacaag	atcatgaaagccaaattgatagtc	RT-PCR of *sufB*
SufS	acagtcattactgaaattcctgtta	tgtctatagtaatcattcaaacaag	RT-PCR of *sufS*
SufD-rt	tatgcccaaatcgctgaagaactc	aagcgtttttcttgtagccaagcag	RT-PCR of *sufD*
SufC-rt	agacaagaagaaagtccaattggac	ctcgctcatcaacttccatttccaa	RT-PCR of *sufC*

Note: Sequences underlined are restriction sites engineered for cloning.
