# Supplementary material for: Streptococcus mutans Lacking sufCDSUB Is Viable, but Displays Major Defects in Growth, Stress Tolerance Responses and Biofilm Formation
Source: Front Microbiol. 2021 Jun 24;12:671533. doi: 10.3389/fmicb.2021.671533 (PMC8264796; doi:10.3389/fmicb.2021.671533)
Supplement: Supplementary file 3 [file Table_2.pdf]

Table S2.

(A) Doubling times when grown in regular BHI and under BHI adjusted to pH 6

**Strain**	**Regular BHI**	**BHI adjusted to pH 6**
UA159	1.81 (± 0.05) hours	3.96 (± 0.75) hours
Δ*suf*	2.83 (± 0.25) hours*	4.68 (± 0.15) hours*
Δ*sufC*	2.36 (± 0.03) hours,	2.88 (±0.03) hours
Δ*sufD*	2.37 (± 0.03) hours,	3.17 (±0.03) hours
Δ*sufS*	2.27 (± 0.02) hours,	3.87 (± 0.36) hours
Δ*sufU*	2.23 (± 0.02) hours	3.70 (± 0.25) hours
Δ*sufB*	2.36 (± 0.01) hours	5.36 (± 0.58) hours*
sufC	2.40 (± 0.04) hours	2.83 (±0.02) hours
Δ*suf*+	1.82 (± 0.05) hours	3.92 (± 0.22) hours

* $P<0.05$

(B) Doubling times when grown in the absence of branched-chain amino acids

**Strain**	**FMC**	**FMC-isoleucine**	**FMC-leucine**	**FMC-glutamate/glutamine**
UA159	1.53 (±0.03) hours	1.44 (±0.01) hours	1.63 (±0.06) hours	1.47 (±0.01) hours
Δ*suf*	2.03 (±0.02) hours*	9.29 (±0.70) hours*	8.33 (±0.12) hours*	2.25 (±0.03) hours*

* $P<0.05$
